# Poly(hydromethylsiloxane) Networks Functionalized by N-allylaniline

**DOI:** 10.3390/ijms26146700

**Published:** 2025-07-12

**Authors:** Anita Wysopal, Maria Owińska, Ewa Stodolak-Zych, Mariusz Gackowski, Magdalena Hasik

**Affiliations:** 1 Faculty of Materials Science and Ceramics, AGH University of Krakow, 30-059 Kraków, Poland; 2 Jerzy Haber Institute of Catalysis and Surface Chemistry, Polish Academy of Sciences, 30-239 Kraków, Poland; mariusz.gackowski@ikifp.edu.pl

**Keywords:** polysiloxanes, poly(methylhydrosiloxane), amine-functionalized polysiloxanes, antibacterial polysiloxanes, antibacterial polymers

## Abstract

Polymers containing biocidal moieties (e.g., amino or ammonium groups) are considered promising materials that can help combat the growing resistance of pathogens to commonly used antimicrobials. Searching for new polymeric biocides, in this work, non-porous and porous poly(hydromethylsiloxane) (PHMS) networks were prepared and post-functionalized by N-allylaniline (Naa). Non-porous networks were obtained by cross-linking PHMS in the bulk and porous—in W/O high-internal-phase emulsion (HIPE). Linear divinyldisiloxane (M_2_^Vi^) or cyclic tetravinyltetrasiloxane (D_4_^Vi^) were used as cross-linkers. Studies confirmed the expected non-porous and open macroporous microstructure of the initial networks. They also showed that functionalization by Naa was more efficient for the non-porous networks that swelled to lower extents in toluene and contained higher amounts of Si-H groups than the porous ones. In the reactions with benzyl chloride or 1-bromoctane, some amino groups present in these materials were transformed to ammonium groups. It was found that activity against Gram-positive *S. aureus* and Gram-negative *E. coli* bacteria depended on the functionalization degree, cross-linking level and the microstructure of the modified materials.

## 1. Introduction

Compounds containing amino groups in their molecules play important roles in nature and in chemistry. Amino acids, as protein building blocks, or nucleic acids, as genetic information encoders/transmitters, are essential for living organisms. In organic chemistry, amines are nucleophiles that react with a range of electrophilic agents to give various substances or bases that capture reaction acidic side products [1]. In coordination chemistry, amines are electron-donating ligands for metal cations or atoms [2]. In polymer chemistry, amines serve as monomers [3], catalysts [4] for the syntheses of macromolecular compounds or as polymer cross-linking agents [5]. Chemical processes involving amines are widely applied in both laboratory practice and industry.

Among amine-derived compounds, quaternary ammonium salts (QAS or quats) constitute a special class. Represented by the general formula [R_4_N^+^]A^−^ (where R—organic moiety, often alkyl or substituted alkyl group, A—halogen atom or hydroxyl group), they are amphiphilic compounds whose physico-chemical properties can be controlled by the type of the organic group and by the type of anion. QAS with long alkyl chains in their molecules, e.g., hexadecyltrimethylammonium bromide/chloride, are cationic surfactants added to cosmetic products [6]. They also play the role of templates in the synthesis of ordered mesoporous silica [7]. Alkylammonium halides (e.g., benzyltriethylammonium chloride), as phase transfer catalysts, facilitate the environmentally benign preparation of a wide range of organic compounds in immiscible water-organic solvent media [8]. Moreover, well-documented antimicrobial properties of QAS allow their applications as disinfectants, antiseptics or preservatives [9]. The mechanism of their action involves the interaction of the positively charged ammonium group with the negatively charged microbial cell membrane followed by penetration of the hydrophobic part of QAS through the hydrophobic membrane core, which finally leads to the microbe’s death [10].

Currently, the growing resistance of pathogens to biocides is a serious problem. One of the possible ways to address it that has been explored extensively in recent years [11] is the synthesis of polymers chemically modified by the moieties that show antimicrobial properties. The high density of such groups attached to the polymer chains leads to enhanced activity with respect to the corresponding low-molecular-weight biocides [12]. Their covalent binding ensures high stability and allows long-term use of the systems without leaching of toxic, low-molecular-weight bioactive compounds to the environment. Additionally, preparation of the functionalized polymers is relatively easy and a wide range of macromolecular compounds and modifying groups can be involved, which holds promise for the development of new, efficient antimicrobial agents.

Amino and ammonium moiety-containing polymers are an important class of macromolecular biocides. Bioactivity of common organic macromolecules, such as methacrylate homo- and copolymers [13,14,15,16], polycarbonates [17,18,19], polystyrenes [12,20,21] and polyurethanes [22,23,24] functionalized by these groups, was studied. As a result, distinct relationships between the chemical structure of the modified compounds and their antimicrobial properties were established. Most importantly, hydrophobic/hydrophilic balance determined either by the chemical structure of the ammonium group or that of the macromolecule was found to be the main factor governing the bioactivity of polymers [13,14,16,17,18,19,21]. Interestingly, high antibacterial activity of the compounds containing primary ammonium (PA) [13,18,19] or tertiary ammonium (TA) [13] moieties was demonstrated. The high activity of polymers with introduced quaternary ammonium (QA) groups required the presence of long alkyl chains in the biocidal cation [13,17,21].

It is worth noting that the polymers modified by amino/ammonium groups were prepared by polymerization of a functionalized monomer [12,13,14,15,16,18,19,22,23,24] or by post-functionalization of the previously synthesized polymer containing appropriate reactive groups [17,20,21]. Using these methods, antibacterial powders [13,14,15,16,17,18], oils [19], coatings [20] or foams [22,23,24] were obtained.

In our previous work, we have shown that post-functionalization is a convenient way to incorporate amino side groups into poly(hydromethylsiloxane) (PHMS), i.e., the polymer with the inorganic backbone formed by Si-O bonds [25]. PHMS contains reactive Si-H moieties, which—in the presence of a catalyst—readily add to multiple carbon–carbon or carbon–heteroatom bonds; such processes are often referred to as hydrosilylation [26]. In our studies, PMHS was applied as the hydrosilylating agent for *N*-allyl compounds: *N*-allylaniline (Naa), *N*-allylcyclohexylamine (Nach), *N*-allylpiperidine (Nap) and 4-vinylpyridine (PVP) [25]. After the reactions, linear PHMS modified by the amino moieties originating from Naa, Nach or Nap was obtained, while the functionalization of PHMS by PVP did not occur [25].

Due to our experience in the studies of polysiloxane networks, also including those prepared from PHMS [27,28], in the present study, we decided to verify if it is possible to introduce amino groups into the cross-linked PHMS. We synthesized PHMS networks using two siloxane cross-linking agents (linear and cyclic) of different microstructures (non-porous and porous). Then, the networks were functionalized via the reaction with Naa followed by treatment with benzyl chloride (BnCl) or octyl bromide (OcBr) aimed at converting the amino groups incorporated into the systems to the ammonium groups. The materials obtained at each step were thoroughly characterized using a range of experimental techniques. This enabled us to conclude on the influence of the chemical structure and the microstructure of PHMS networks on their functionalization by Naa. Finally, antibacterial properties of the modified materials were tested.

It should be pointed out that to the best of our knowledge, cross-linked PHMS functionalized by Naa has never been investigated before. The work published on antimicrobial, amino or ammonium group-containing polysiloxanes concerned mainly linear polymers bearing reactive units (3-chloropropyl [29,30,31], 3-mercaptopropyl [31], Si-H [32]) that allowed their post-functionalization. After reactions with N,N-dimethyloctylamine [29], N,N-dimethyldodecylamine [30], BnCl [32] or *t*-butylamine 2-(t-butylaminoethyl) methacrylate [31], these polymers were modified by QA [29,30,32] or secondary ammonium/secondary amino [31] moieties and were active against a range of bacteria strains. Quite recently, thiolene “click reaction” between vinyl groups present in random or block siloxane copolymers and cysteamine (i.e., 2-aminoethanethiol) hydrochloride produced polymers with primary ammonium side groups; they were efficient in reducing the growth of several strains of Gram-positive and Gram-negative bacteria as well as phytopathogenic fungi [33].

So far, significantly less effort has been devoted to the antimicrobial cross-linked polysiloxanes with introduced amino or ammonium substituents. Silanol group-terminated siloxane copolymers modified by QA units were cross-linked or *co*-cross-linked with a commercial silicone elastomer using tetraethoxysilane in the presence of a dibutyltin dilaurate catalyst [34]. Both types of materials exhibited high antibacterial activity, even though for the elastomer-containing materials, longer contact times were needed to obtain a high bacteria growth inhibition effect [34]. Silanol-terminated siloxane copolymers containing QA groups were also cross-linked with a commercial polyisocyanate to give antibacterial coatings [30].

Antimicrobial cross-linked polysiloxanes are attractive since they are especially well-suited fillers for siloxane polymers, commonly known as silicones, which are biomaterials widely used in the fabrication of medical devices (urinary catheters, drains, etc.) or implants [35]. With our investigations, we intended to add some new knowledge into the field of antimicrobial polysiloxanes. Their advantage—as compared to the other polymers concerning this topic published in the literature—is that PHMS is an inexpensive, commercially available polymer. Moreover, in our work, for both PHMS cross-linking and modification of its networks by Naa, a well-known, simple hydrosilylation process was applied.

## 2. Results and Discussion

### 2.1. Starting Materials

**Figure 1:**
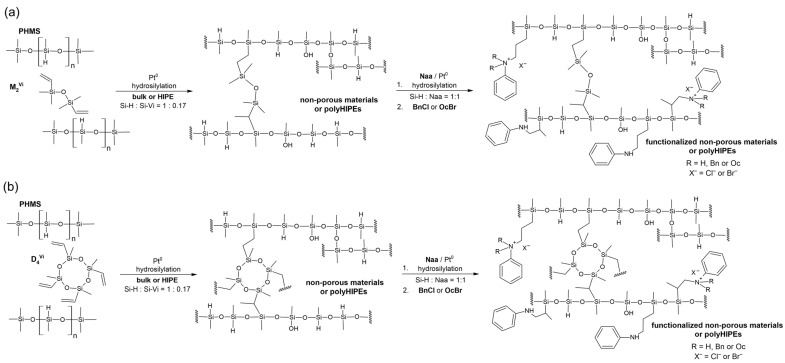
Strategy for the preparation of the materials studied in the work with the use of (**a**) M_2_^Vi^ and (**b**) D_4_^Vi^ as a cross-linking agent.

In the work, four types of PHMS-based materials, namely non-porous (NP_M and NP_D) and porous (P_M and P_D) materials, were functionalized by the moieties originating from Naa. The starting (i.e., subjected to functionalization) materials were obtained by cross-linking of the polymer with a linear, difunctional or cyclic, tetrafunctional vinylsiloxane, M_2_^Vi^ or D_4_^Vi^, respectively. The cross-linking process involved hydrosilylation of the vinyl groups in the cross-linker by the Si-H groups of PHMS catalyzed by the Pt complex (the so-called Karstedt catalyst). It was carried out either in the bulk or in water-in-oil high internal phase emulsion (HIPE) to prepare non-porous or porous polyHIPE materials (polyHIPEs), respectively. Hydrosilylation was also applied in the functionalization of the obtained networks. The overall strategy for the preparation of the starting and functionalized materials adopted in the work is schematically depicted in Figure 1.

As previously mentioned (Section 1), the studies were primarily aimed at evaluating the impact of the microstructure and the chemical structure of the PHMS-derived materials on their functionalization by Naa. Therefore, both parameters were carefully examined. The microstructure of all the prepared materials was determined using SEM, while their chemical structure was determined by FTIR and ^29^Si MAS-NMR spectroscopies (Section 3.6). Additionally, in order to estimate differences in cross-linking degrees of PHMS in the obtained networks, equilibrium swelling measurements were performed (Section 3.6). Cross-linking degrees seemed to be also an important factor influencing subsequent functionalization by Naa of both non-porous and porous materials. This is because in highly cross-linked networks, some of the Si-H groups, necessary for the reaction, may be inaccessible for Naa molecules, which would hinder functionalization. Moreover, polymer cross-linking degrees could affect the microstructure of the porous materials and thus indirectly influence their functionalization by Naa. The dependence of the microstructure on the polymer cross-linking degree was evident in organic polymer-based polyHIPEs [36,37] as well as in polyHIPEs synthesized from poly(methylvinylsiloxane) [38]. In contrast, the microstructure was not related to the amount of the cross-linker (D_4_^Vi^) applied in the preparation of PHMS-based polyHIPEs [39]. However, the lowest amount of D_4_^Vi^ used in Ref. [39] (molar ratio of Si-H: Si-Vi groups equal to 1:0.66) was almost quadruple of that employed in the present work (Si-H:Si-Vi groups’ molar ratio equal to 1:0.17, Section 3.2). Hence, it was of interest to check if the proper polyHIPE microstructure could be developed at such a low amount of a cross-linker as compared to PHMS. Finally, to complete their characterization, skeletal density of all the materials and apparent density of the porous ones were determined (Section 3.6). Based on skeletal and apparent density, total porosity of the fabricated polyHIPEs was calculated (Section 3.6).

**Figure 2:**
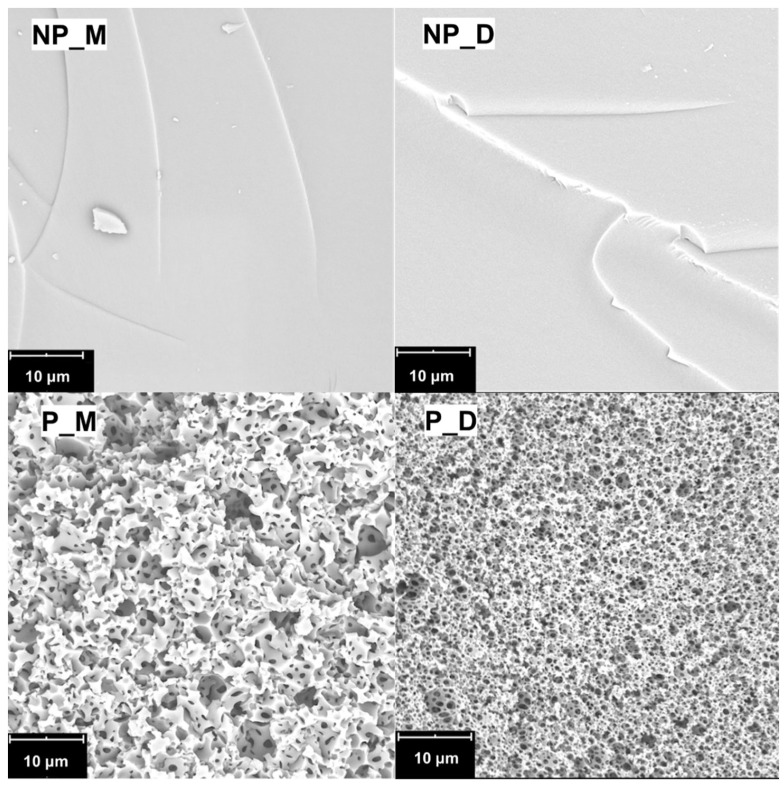
SEM images of non-porous materials (NP_M and NP_D) and polyHIPEs (P_M and P_D) prepared in the work.

SEM images (Figure 2) showed that, as expected, the NP_M and NP_D materials, prepared in the bulk, contained no pores and had a smooth surface. The microstructure of the materials synthesized in HIPE (Figure 2) was typical of that characteristic for polyHIPEs [40,41]. Thus, large macropores, often called voids [40] interconnected by smaller ones, referred to as windows [40] were visible. Hence, the P_M and P_D materials exhibited open porosity, a feature favorable for applications in which transport of chemicals, nutrients, etc., within a material is required. Differences in the microstructure of the NP_M, NP_D and P_M, P_D materials were also supposed to lead—after modification with biocidal moieties—to various kinds of antibacterials.

According to SEM, voids and windows of higher maximum diameter existed in the P_M than in the P_D sample (Table 1). Interestingly, voids and windows in P_M and P_D materials were smaller than those in the PHMS-derived polyHIPEs prepared by us previously [39]. This could be due to lower water content in HIPEs used in the present studies (74 wt%, Section 3.2) compared to in the previous (82 wt% [39]) studies. Pore sizes in polyHIPEs are known to decrease as the fraction of water in the emulsion applied for their preparation decreases [37,42].

Distribution plots constructed based on quantitative SEM image analysis performed using ImageJ software, version 1,54k (Section 3.6) showed that the diameters of voids in the P_M were higher than in the P_D polyHIPE, whereas diameters of windows were similar in both materials (Figure S1, Table 1). These conclusions were confirmed by a formal statistical Mann–Whitney U-test (Section 3.6), which gave *p* < 0.001 and *p* = 0.95 for diameters of voids and windows, respectively (*p* is the probability that there is no difference in the analyzed data). As a consequence, the P_M sample was characterized by higher mean and median void diameters while maintaining the same values for the windows as in the P_D sample (Table 1). Thus, SEM studies pointed to the influence of the structure of the cross-linking agent (linear/cyclic, difunctional/tetrafunctional) on the microstructure of the obtained porous materials.

In addition, SEM results unequivocally proved that a low amount of the cross-linker with respect to PHMS in HIPE did not preclude the formation of the proper polyHIPE microstructure. This confirmed that, as established in our earlier work [39], in PHMS-based polyHIPEs, this microstructure was developed independently of the reactive groups’ molar ratio in HIPE during their preparation. Since the characteristic macroporous polyHIPE structure reflects the dispersion of internal phase droplets in HIPE around which the polymer network is created [40,41], it can be generated only in a stable emulsion. HIPEs whose composition changes upon polymer cross-linking are rather unstable; therefore, network formation has to occur fast before the emulsion collapses. In the present study, a high rate of network formation via hydrosilylation was unlikely due to the very low amount of the vinylsiloxanes used. For this reason, a different cross-linking process must have taken place. It could have involved condensation of Si-OH groups resulting from the hydrolysis of Si-H moieties present in PHMS. Hydrolysis of Si-H groups is catalyzed by metals [43], including Pt [44]. Pt catalysts of the hydrosilylation reaction simultaneously catalyze hydrolysis of Si-H groups [45].

As already stated, spectroscopic (FTIR and ^29^Si MAS-NMR) investigations were applied to determine the chemical structure of the synthesized networks. In spectra analysis, particular attention was paid to establishing the presence of Si-H groups as they were necessary for further functionalization of the materials carried out by hydrosilylation of Naa (Figure 1). In view of SEM results, clarifying whether hydrolysis of Si-H to Si-OH groups and condensation of the latter took place in HIPE was an important point in the spectroscopic studies of P_M and P_D materials.

**Figure 3:**
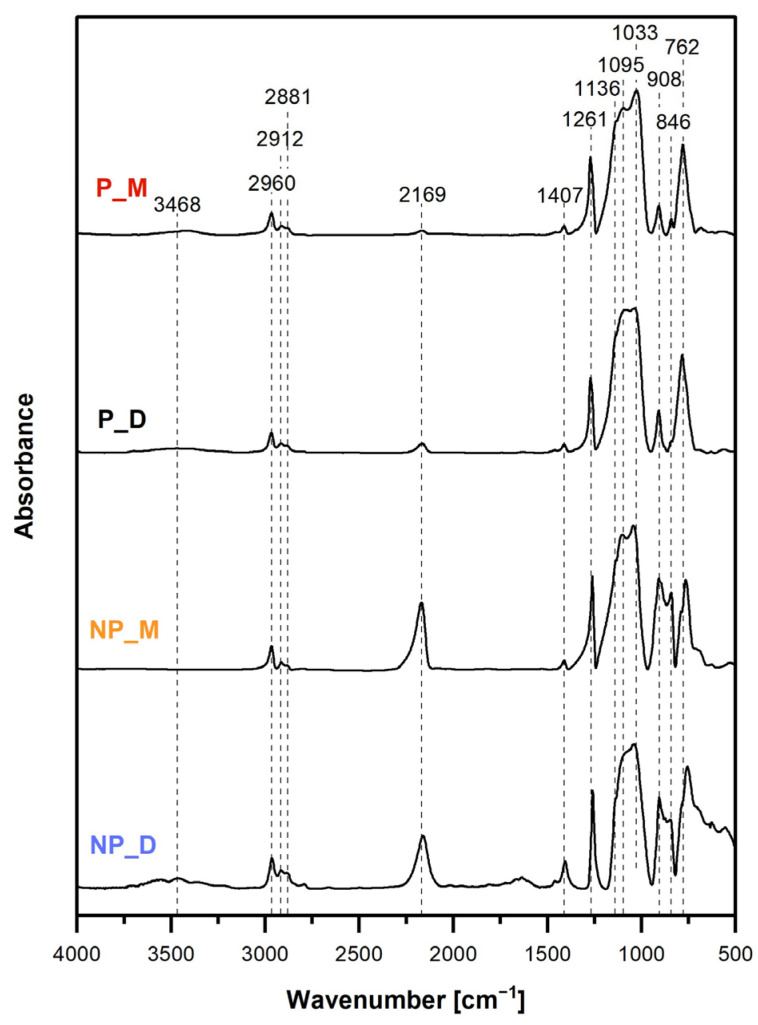
FTIR spectra of the starting materials.

FTIR spectra (Figure 3) confirmed that all the prepared materials showed the structure expected for the products of M_2_^Vi^ or D_4_^Vi^ hydrosilylation with PHMS. Thus, they contained Si–O bonds as well as C–H bonds in methyl (Si–CH_3_) and ethylene (–CH_2_CH_2_–) groups. These bonds gave rise to the characteristic bands at 1033 cm^−1^ (symmetric stretching vibrations of Si–O–Si [46]), 762 cm^−1^, 1261 cm^−1^, 2960 cm^−1^ (C–H rocking combined with Si–C stretching, C–H symmetric bending, C–H asymmetric stretching modes, respectively, in Si–CH_3_ [46]), 1136 cm^−1^, 2881 cm^−1^ and 2912 cm^−1^ (C–H bending, C–H symmetric and antisymmetric stretching modes, respectively, in –CH_2_CH_2_– bridges [46]).

Moreover, FTIR spectra signified the required existence of Si-H groups in the networks. This was proved by the bands at 908 cm^−1^ (Si-H bending vibrations) and 2169 cm^−1^ (Si-H stretching vibrations [46]) seen in the spectra of all the materials (Figure 3). However, the intensities of these bands in the spectra of the NP_M and NP_D samples were significantly higher than in those of P_M and P_D samples. This indicated higher conversion degrees of Si-H groups occurring in HIPE than in the bulk. Quantitative FTIR spectra analysis in which the ratios of the integral intensities of the band due to the Si–H groups at 2169 cm^−1^ and the band ascribed to the C–H bonds in Si–CH_3_ groups at 1261 cm^−1^ were calculated (Section 3.6) showed that conversion degrees of Si–H groups for the porous materials were 1.5 times higher than those found for the non-porous ones prepared with the same cross-linker (P_M: 94.8% vs. NP_M: 62.0% and P_D: 88.5% vs. NP_D: 58.3%, Table 2). Since in all the reactions, the same molar ratio of groups involved in hydrosilylation was used (Si-H:Si-Vi =1:0.17, Section 3.2), it could be concluded that in HIPEs, Si-H moieties participated in side processes. Hydrolysis was the first process to be considered because of the high water content in the emulsions and because it also occurred in our previous studies [28,38,39].

FTIR spectra of the P_M and P_D materials prepared in HIPEs (Figure 3) contained a broad band centered at 3468 cm^−1^, which could be ascribed to Si–OH groups (O–H stretching mode [46]). Its low intensities suggested low amounts of Si-OH moieties in these systems, possibly brought about by their fast condensation accompanied by the formation of siloxane bonds in new silsesquioxane (-SiO_3_) units (Figure 1), not detected in the FTIR spectra. The band due to Si-OH bonds was not visible in the spectrum of the NP_M material, but—quite surprisingly—it was distinct in the spectrum of the NP_D sample (Figure 3). Hence, according to FTIR spectroscopy, hydrolysis of Si-H groups to Si-OH groups proceeded in HIPEs during the preparation of the P_M and P_D materials and also in the bulk when D_4_^Vi^ was used as a cross-linker.

**Figure 4:**
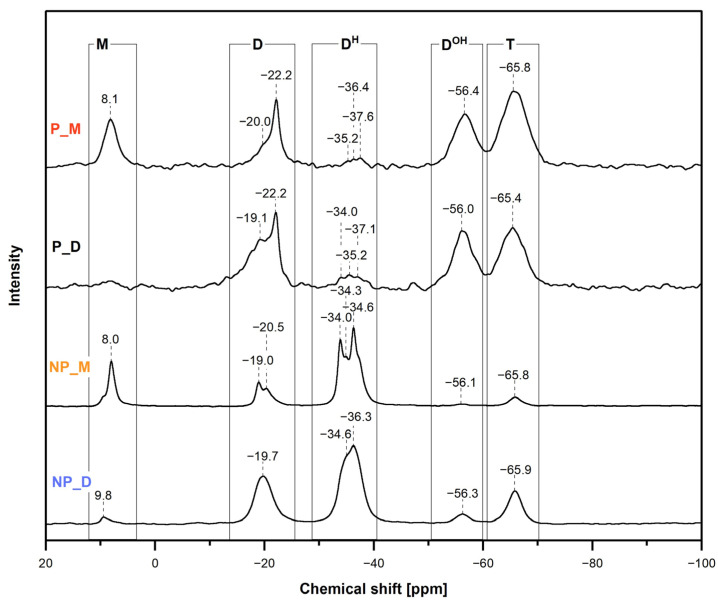
^29^Si MAS-NMR spectra of the starting materials.

^29^Si MAS-NMR spectroscopic investigations (Section 3.6) supported the conclusions drawn from the FTIR spectra and provided some complementary information on the chemical structure of the prepared materials. Thus, the ^29^Si MAS-NMR (Figure 4) in line with the FTIR spectra showed that the studied materials were formed by hydrosilylation of the cross-linkers by PHMS and that they contained Si-H and Si-OH groups. In addition, ^29^Si MAS-NMR corroborated the occurrence of condensation of Si-OH groups, which was not shown by FTIR spectroscopy.

In the ^29^Si MAS-NMR spectra of the materials obtained using M_2_^Vi^ (NP_M and P_M samples), hydrosilylation was manifested by the presence of signals at chemical shift values *δ* = 8.0/8.1 ppm and in the range between −19.0 and −22.2 ppm (Figure 4). The signal at *δ* = 8.0/8.1 ppm could be assigned to the [SiO(CH_3_)_2_(CH_2_CH_2_)] units resulting from hydrosilylation (Figure 1) as well as to the [SiO(CH_3_)_3_] terminal groups in PHMS (Section 3.1), i.e., to the so-called M units present in siloxane compounds [47]. However, as could be judged by the shares of this signal in the deconvoluted spectra of the P_M and NP_M materials (Table 2), the one corresponding to the [SiO(CH_3_)_2_(CH_2_CH_2_)] units was its only component. A set of signals in the range between −19.0 and −22.2 ppm in turn originated from the [SiO_2_(CH_3_)(CH_2_CH_2_)] or [SiO_2_(CH_3_)(CH(CH_3_))] units in various chemical environments, i.e., the so-called D units of siloxane compounds [48,49] formed upon hydrosilylation (Figure 1). The shares of the M and D signals in the spectra of the NP_M and P_M materials were equal (Table 2) since hydrosilylation of M_2_^Vi^ by PHMS yields an equal number of [SiO_2_(CH_3_)(CH_2_CH_2_)]/[SiO_2_(CH_3_)(CH(CH_3_))] and [SiO(CH_3_)_2_(CH_2_CH_2_)] units (Figure 1).

Upon hydrosilylation of D_4_^Vi^ by PHMS, exclusively D units ([SiO_2_(CH_3_)(CH_2_CH_2_)] or [SiO_2_(CH_3_)(CH(CH_3_))]) were generated (Figure 1). Their presence in the NP_D and P_D materials was evidenced by broad signals with maxima at *δ* = −19.1 or −19.7 and −22.2 ppm in the ^29^Si MAS-NMR spectra (Figure 4). Shares of these signals, obtained after spectra deconvolution, were ca. double of those found in the spectra of the networks prepared with M_2_^Vi^ (Table 2). Thus, PHMS hydrosilylation degrees were similar in all the systems (15–18%). It is worth noting that they were close to that expected for the reactive groups’ molar ratio applied in the experiments (Si-H:Si-Vi = 0.17, Section 3.2).

Signals due to the [SiO_2_(CH_3_)H] [47], often referred to as D^H^ units, were visible at *δ* = −34.0–−37.6 ppm in all the recorded ^29^Si MAS-NMR spectra (Figure 4). Their shares were markedly higher in the spectra of the non-porous materials than in those of porous materials (Table 2). This indicated, in agreement with FTIR results, that the NP_M and NP_D samples contained significantly more Si-H groups than the P_M and P_D polyHIPEs. According to ^29^Si MAS-NMR, the highest fraction of [SiO_2_(CH_3_)H] units contained the NP_M sample (Table 2).

Hydrolysis of some Si-H followed by condensation of the resulting Si-OH groups to silsesquioxane groups was demonstrated by the appearance in all the ^29^Si MAS-NMR spectra of the signals at *δ* = −56.0–−56.4 ppm and −65.4–−65.9 ppm (Figure 4). They could be assigned to the [SiO_2_(CH_3_)OH], i.e., D^OH^ units and [SiO_3_(CH_3_)], called T units, respectively [47], occurring in the networks (Figure 1). Shares of these signals in the spectra of the porous materials were higher than in those of the non-porous ones (Table 2). Thus, as expected, hydrolysis/condensation processes were more pronounced in HIPEs than in the bulk. Hydrolysis in the bulk must have been caused by water contained in the chemicals, which were applied in the experiments without prior purification, or water from the environment (experiments were performed in the flow of Ar, not in a dry box). Out of these two possibilities, the latter seemed more probable as only cross-linkers were not dried before use in the syntheses of the non-porous materials (Section 3.1). However, cross-linkers did not contain water, which was verified by their FTIR spectra. Nonetheless, ^29^Si MAS-NMR in concert with FTIR spectroscopy showed a higher hydrolysis degree in the NP_D than in the NP_M sample (Table 2).

Silsesquioxane (T) units established by ^29^Si MAS-NMR spectroscopy could constitute additional (with respect to those formed by hydrosilylation) cross-links in the prepared networks. Equilibrium swelling measurements in toluene were performed (Section 3.6) to explore the effect of the presence of the T groups on cross-link degrees as well as to verify if there were differences in cross-linking degrees in the studied materials.

Experiments revealed that the samples containing more silsesquioxane groups swelled to a lower extent than those of a similar microstructure but with lower T group fractions (P_M vs. P_D and NP_D vs. NP_M materials, Table 2). Consequently, it could be concluded that the condensation of Si-OH groups contributed to the PHMS cross-linking process and led to the growth in cross-linking degrees in the networks.

It was also found that the porous (P_M and P_D) materials swelled more than the non-porous (NP_M and NP_D) materials (Table 2). This indicated higher cross-linking degrees in the non-porous networks. It should be noted at this point that swelling of a non-porous polymer network is directly related to its cross-linking level. Estimation of cross-linking levels in polyHIPEs based on equilibrium swelling measurements is not so straightforward because of the possible sorption of the solvent in the pores [50,51]. Therefore, to compare cross-linking degrees, polyHIPEs of similar porosity should be examined. In the present studies, P_D material could have absorbed more toluene due to its higher total porosity than that of the P_M polyHIPE (89.4% vs. 80.9%, respectively, Table 2). However, the difference in porosity of these samples (porosity ratio equal to 1.1) was significantly lower than the difference in their swelling extents (swelling degree ratio: 1.88). Therefore, it could be supposed that various swelling levels of the P_D and P_M networks were mainly attributed to their various cross-linking degrees.

For the non-porous materials, the relationships between cross-linking degrees determined by equilibrium swelling experiments were confirmed by skeletal density measurements (Section 3.6). As expected, the NP_M sample of a higher cross-linking degree was characterized by a lower skeletal density, while the NP_D sample of a lower cross-linking degree was characterized by a higher skeletal density (Table 2). In contrast, the relationships between skeletal densities and cross-linking levels of the studied polyHIPEs were not as anticipated. The P_D sample of a lower cross-linking level found by swelling measurements showed higher skeletal density than the P_M one in which the cross-linking density was higher (Table 2). However, in the case of the P_M and P_D materials, the apparent density was more important as it was connected with their porous structure. Thus, as expected, the P_M polyHIPE exhibited a higher apparent density because of its lower total porosity, whereas the lower apparent density of the P_D polyHIPE could be explained by its higher total porosity (Table 2).

In summary, studies of the NP_M, NP_D, P_M and P_D materials showed that they differed in their microstructure, contained various amounts of Si-H groups and were characterized by various equilibrium swelling degrees in toluene, a feature related to various cross-linking degrees of the prepared networks but also to the differences in their microstructure.

### 2.2. Functionalized Materials

In the next part of the work, the non-porous (NP_M, NP_D) and the porous (P_M, P_D) materials were functionalized by Naa. The process involved hydrosilylation of the double carbon–carbon bonds in the allyl groups of Naa molecules by Si-H groups remaining in the networks performed in the presence of Karstedt catalyst (Section 3.4, Figure 1). Then, the functionalized materials (NP_M/Naa, NP_D/Naa, P_M/Naa, P_D/Naa) were treated with BnCl or OcBr (Section 3.5) to obtain the materials containing ammonium groups (NP_M/Naa/BnCl, NP_D/Naa/BnCl, P_M/Naa/BnCl, P_D/Naa/BnCl or NP_M/Naa/OcBr, NP_D/Naa/OcBr, P_M/Naa/OcBr, P_D/Naa/OcBr, Figure 1).

As found in the studies (Section 2.1), the starting networks showed different microstructure and equilibrium swelling/cross-linking degrees, which could affect their modification by Naa. Most importantly, however, they differed in the contents of Si-H groups, necessary for the reaction with the amine. Due to hydrolysis, particularly low amounts of Si-H moieties were preserved in the prepared polyHIPEs (P_M and P_D materials). Therefore, after the reactions with Naa, the initial question was whether the modification of the networks had occurred. The answer was provided by elemental (C, H, N) analysis and by FTIR and ^29^Si-MAS NMR spectroscopies (Section 3.6).

Elemental analysis showed that all the materials resulting from the reactions with Naa contained nitrogen (Table 3), which indicated their successful functionalization. However, the amounts of nitrogen in the samples were low and in most cases, they were significantly lower than those calculated based on the stoichiometry of the functionalization process. Expectedly, due to low amounts of Si-H groups remaining in the porous starting networks (Section 2.1), lower contents of nitrogen were found in the P_M/Naa and P_D/Naa than in the NP_M/Naa and NP_D/Naa samples (Table 3).

Among both non-porous and porous materials, the amounts of nitrogen were higher for those that swelled to a lower extent (nitrogen contents: P_M/Naa > P_D/Naa, NP_D/Naa > NP_M/Naa, Table 3; swelling degrees: P_M < P_D, NP_D < NP_M, Table 2). This, quite surprisingly, implied more efficient functionalization in more cross-linked networks. The possible reason for this phenomenon could be that during the reactions with Naa, performed in toluene (Section 3.6), the materials were in a swollen state. Possibly, their spatial and high-volume nature in this state limited contact of the reactive groups, thus lowering functionalization yield. The effect of swelling was noticeably stronger in the porous materials. The P_D/Naa sample, prepared from the P_D polyHIPE whose swelling was almost twice that found for the P_M sample (Table 2), contained three times less nitrogen than the P_M/Naa material (Table 3). In the case of the non-porous networks, a twofold decrease in the extent of swelling was accompanied by a twofold growth in the nitrogen content (Table 2 and Table 3). Since swelling of porous materials was considerably higher than that of the non-porous ones, these findings supported the conclusion on the adverse influence of swelling on the modification by Naa of the cross-linked PHMS studied in the work. Thus, these results suggest that controlling cross-linking density and porosity could be an effective strategy to optimize Naa functionalization efficiency.

Additionally, by elemental analysis, higher contents of carbon in the functionalized non-porous materials compared to porous materials (NP_M/Naa, NP_D/Naa vs. P_M/Naa, P_D/Naa, Table 3) were determined. This was in line with the higher contents of Naa groups in the former because incorporation of Naa moieties comprising a phenyl ring should lead to increased carbon amounts in the networks.

**Figure 5:**
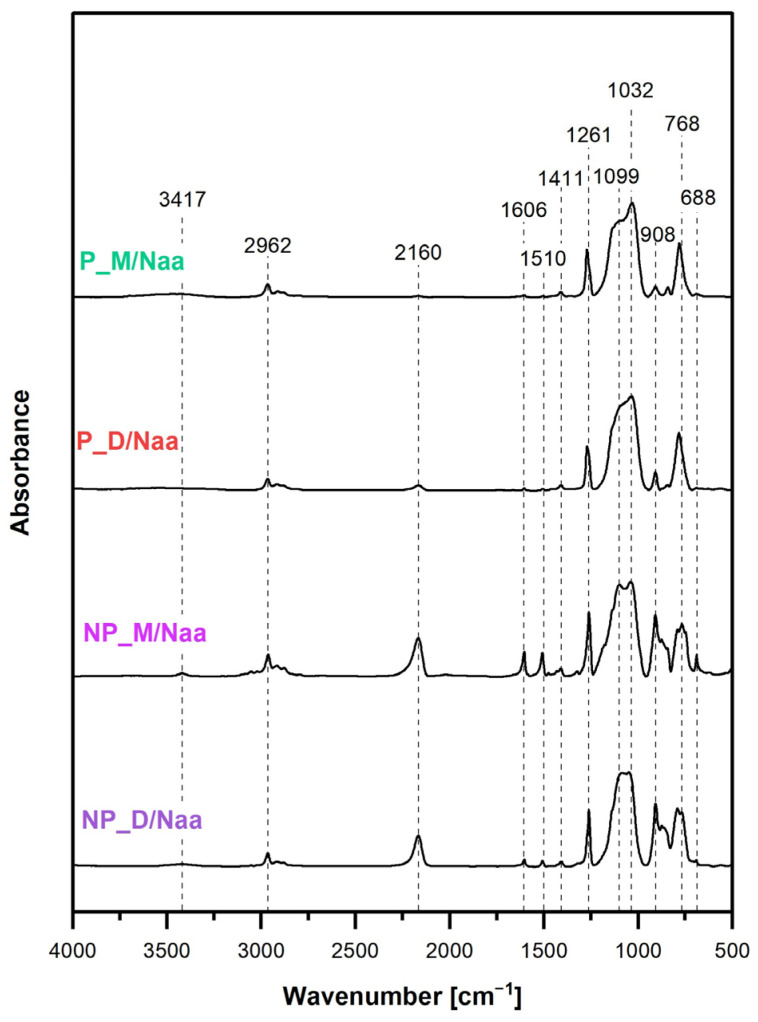
FTIR spectra of the studied porous and non-porous materials functionalized with Naa.

FTIR spectra of the materials treated with Naa (Figure 5) showed decrease in intensities of the band at 2160 cm^−1^, corresponding to stretching vibrations of Si-H groups with respect to those measured before functionalization (Figure 3). This confirmed that Si-H groups were involved in the reaction. According to quantitative FTIR spectra analysis (Section 3.6), transformation degrees of Si-H groups were low: 3.5% and 4.3% for the porous materials and 7.2% and 8% for the non-porous ones (Table 3). Higher transformation degrees of Si-H groups found by FTIR spectroscopy agreed with the higher nitrogen contents in the non-porous samples determined by elemental analysis (Table 3).

Presence of Naa moieties was demonstrated by weak bands at 3417 cm^−1^, 1606 cm^−1^ and 1510 cm^−1^ in the FTIR spectra of NP_M/Naa and NP_D/Naa samples (Figure 5). The former band could be unambiguously attributed to N–H stretching vibrations, whereas the band at 1606 cm^−1^ was likely the superposition of a weak band due to C = C stretching vibrations of the aromatic ring and an intense band ascribed to N–H deformational vibrations [46]. These bands were not seen in the spectra of the P_M/Naa and P_D/Naa materials, which could be explained by their low functionalization levels.

Hydrosilylation of Naa by Si-H groups preserved in the studied networks should be accompanied by the formation of new [SiO_2_(CH_3_)(CH_2_CH_2_CH_2_)]/ [SiO_2_(CH_3_)(CH(CH_3_)CH_2_)], i.e., D units in the reaction products (Figure 1). Simultaneously, the contents of [SiO_2_(CH_3_)H], i.e., D^H^ units, should decrease with respect to those in the starting materials. It is worth noting here that even though usually the *anti-*Markovnikov product with linear hydrocarbon group predominates in hydrosilylation of double carbon–carbon bonds conducted over Pt catalysts, in many cases, the branched, Markovnikov adduct is formed as well [45]. Its presence was proved in the products of hydrosilylation of Naa by linear PHMS [25] and N,N-dimethylallylamine by copolymers containing hydromethylsiloxane and dimethylsiloxane groups [32] catalyzed by the Karstedt complex. Hence, the generation of [SiO_2_(CH_3_)(CH(CH_3_)CH_2_)] units in this work, when the cross-linked PHMS was used in the reaction with Naa, could not be excluded.

**Figure 6:**
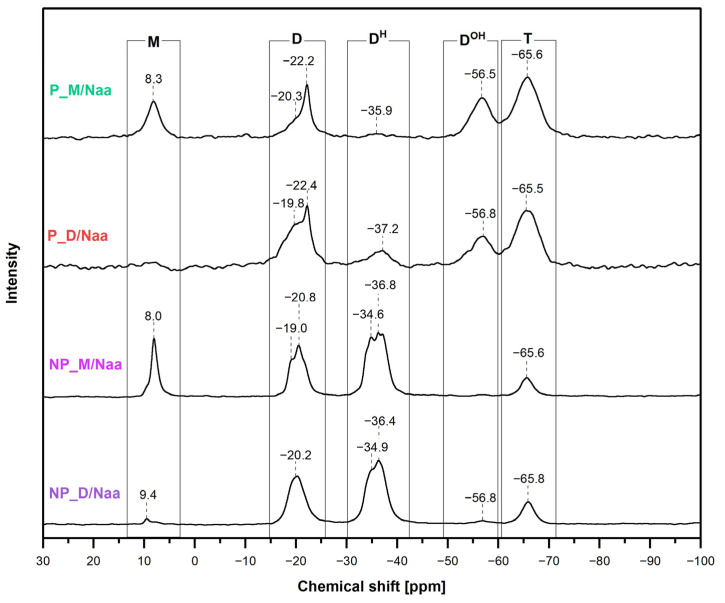
^29^Si MAS-NMR spectra of the porous and non- porous materials functionalized with Naa.

Comparison of the ^29^Si MAS-NMR spectra of the functionalized samples (Figure 6) with those of the initial ones (Figure 4) revealed that all expected changes were observed for the P_M/Naa, NP_M/Naa and NP_D/Naa materials (Table 3). The highest growth in the share of D units and the highest decrease in the D^H^ ones took place during modification of the NP_M material, which—according to ^29^Si MAS-NMR spectroscopy—contained the highest amount of Si-H groups (Table 2). Amounts of D units in the other systems grew to lower extents (1–5%, Table 3). A small decrease in D^H^ unit fractions (1–3%, Table 3) occurred after treatment with Naa of NP_D and P_M networks. Hence, ^29^Si MAS-NMR spectroscopy confirmed that modification of the studied PHMS networks via hydrosilylation of Naa took place, but its degrees were low. The highest one, indicated by the most significant changes in the spectra, was in the NP_M/Naa sample even though it contained less nitrogen found by elemental analysis than the NP_D/Naa material (Table 3). This suggested that in the NP_D/Naa network, unreacted amine remained. Possibly, it was trapped in the bulk of the swollen material after the reaction. It should be noted that, as consistently shown by swelling experiments and ^29^Si MAS-NMR spectra (Table 2), among the non-porous networks subjected to functionalization, the NP_D was characterized by a higher cross-linking degree than the NP_M one. Therefore, it could have been difficult to remove Naa from the NP_D/Naa sample. Additional experiments would be necessary to find out if the presence of unreacted amine in the functionalized cross-linked PHMS can be avoided. For example, modification by Naa could be performed in the solvents in which the polymer networks do not swell. Alternatively, cross-linking of the previously functionalized linear PHMS could be considered. Such studies were, however, beyond the scope of the present work. They will be our future goals.

Furthermore, ^29^Si MAS-NMR spectroscopy showed that side reactions proceeded during functionalization of the studied materials as well. The most prominent was condensation of Si-OH groups manifested by the decrease in the shares of the signals due to D^OH^ units in all the spectra after functionalization with a concomitant increase in the fractions due to T units, evident for the P_D/Naa and NP_M/Naa materials (Table 3). This is understood since amines, as bases, are catalysts of silanol condensation [52]. Additionally, a higher decrease in the amount of D^H^ units than the growth of the amount of D units (Table 3) pointed to the occurrence of hydrolysis of some Si-H groups in the reaction of NP_M network with Naa. The decrease in the amount of T units (NP_D/Naa material, Table 3) or the increase in the amount of D^H^ units (P_D/Naa material, Table 3) indicated occurrence of other side processes. Overall, ^29^Si MAS-NMR spectroscopy reflected the complexity of the studied systems in which various chemical transformations took place.

According to SEM (Figure S2), the microstructure of the starting materials was preserved after functionalization. The surface of the NP_M/Naa and NP_D/Naa samples was smooth and non-porous, while on the surfaces of the P_D/Naa and P_M/Naa samples, macropores, voids and windows were seen.

Modification by Naa caused a small increase in equilibrium swelling in toluene of both non-porous and porous materials cross-linked using M_2_^Vi^ (NP_M/Naa vs. NP_M: 1.2-fold, P_M/Naa vs. P_M: 1.3-fold swelling growth, Table 2 and Table 3). Swelling of the NP_D/Naa and P_D/Naa samples was the same as before Naa treatment (Table 2 and Table 3). This implied that side processes taking place during functionalization involved a small decrease in cross-linking degrees of the NP_M and P_M networks, which did not occur in the NP_D and P_D networks. It should be noted, however, that the growth in total porosity contributed to increased swelling of the P_M/Naa sample. As calculated using skeletal and apparent densities (Section 3.6), total porosity increased from 80.9% for the P_M (Table 2) to 92.5% for the P_M/Naa material (Table 3).

Changes in skeletal density of the non-porous samples upon modification by Naa were small and could be correlated with the changes in their equilibrium swelling. Thus, like swelling, the skeletal density of the NP_M/Naa material (Table 3) was higher than that of the NP_M one (Table 2). Similarly, no changes in their swelling led to the practically unchanged skeletal density of the NP_D/Naa (Table 3) with respect to the NP_D sample (Table 2).

Treatment with BnCl or OcBr (Section 3.5, Figure 1) resulted in the conversion of some of the amine groups present in the non-porous materials to ammonium groups, while in the case of the porous networks, this process did not occur or occurred to a very low extent. This was established by N1s XPS spectra of the non-porous and porous samples showing higher functionalization degrees within each group, i.e., NP_M/Naa and P_M/Naa (Table 3) and products of their reactions with BnCl/OcBr. No changes were detected in the FTIR spectra of the BnCl and OcBr-treated materials (Figure S3).

**Figure 7:**
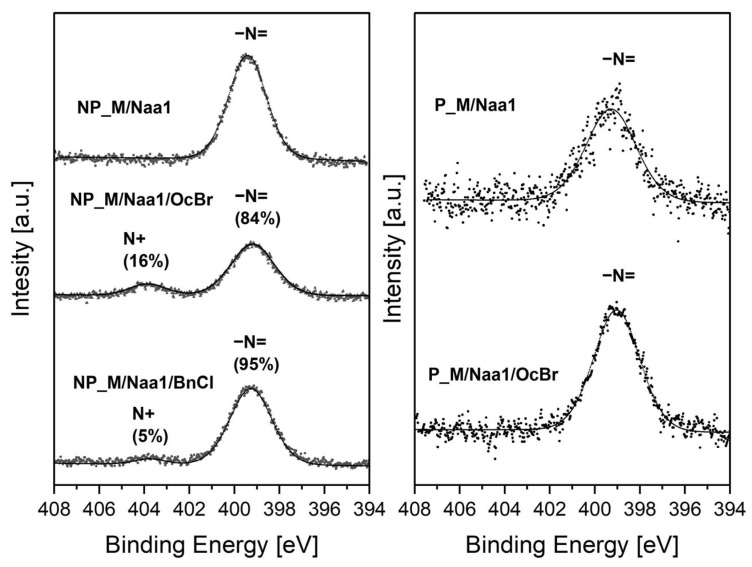
High-resolution N1s XPS spectra of selected studied materials.

The N1s XPS spectra (Figure 7) of the Naa-functionalized materials contained a single maximum at a binding energy (B.E.) equal to 399.3–399.4 eV assigned to nitrogen atoms in amine groups [53]. It was more intensive in the spectrum of the NP_M/Naa than in that of the P_M/Naa sample, proving the higher nitrogen content in the former material that was also found by elemental analysis (Table 3). After treatment with OcBr and BnCl a maximum of low intensity at B.E. = 403.8 eV due to charged nitrogen atoms [53] appeared in the spectra of the non-porous materials (Figure 7). This proved that transformation of a part of amino to ammonium groups took place. As could be judged by the shares of the maximum corresponding to the charged nitrogen atoms in the N1s XPS spectra, the process was more efficient when OcBr was used. In contrast, no change was seen in the spectrum of the Naa-functionalized porous sample treated with OcBr (Figure 7). This implied that amino groups present in this material were either not converted to ammonium ones at all or converted to a low degree, not detected by XPS. It should be mentioned here that incomplete transformation of amino to ammonium groups was also found by N1s XPS for the functionalized polystyrene [20] and polyurethanes [24]. In the latter case, conversion degrees were low (18–21%), in line with our work.

In summary, the studies showed that higher functionalization degrees by Naa were obtained for the non-porous PHMS-derived materials in which higher amounts of Si-H groups were present than in polyHIPEs. For the modification process, less swelling of the networks (i.e., higher cross-linking degree and lower porosity) was advantageous. Some of the amino groups incorporated into the non-porous materials were transformed into ammonium ones via the reaction with BnCl and OcBr; the process was more efficient when OcBr was used. Such a reaction did not occur or occurred to a low extent in the case of Naa-functionalized porous networks.

### 2.3. Antibacterial Properties

Two antibacterial tests were carried out to investigate the antimicrobial properties of porous and non-porous modified PHMS-based materials prepared in the work. The effect of the powdered materials on the bacterial growth kinetics in liquid media was studied and the degree of bacterial reduction by contact with the tested materials was determined (Section 3.7). The kinetic studies, in which no reference was used, allowed us to compare the antibacterial activity of the modified PHMS-derived samples between themselves. In the bacterial reduction investigations, their activity was related to “undisturbed” bacterial growth proceeding without any substance added. Gram-positive *S. aureus* and Gram-negative *E. coli* were selected as bacterial models.

**Figure 8:**
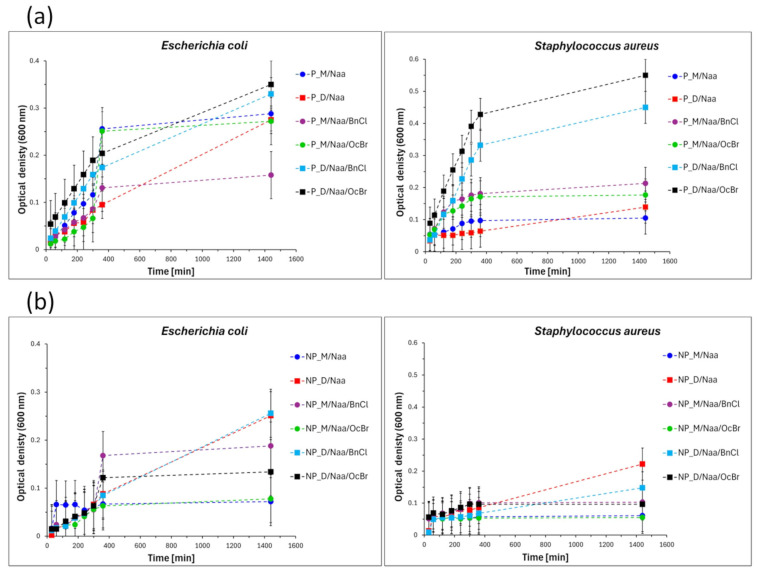
Growth curves of Gram-negative *Escherichia coli* and Gram-positive *Staphylococcus aureus* cultured with modified PHMS materials: (**a**) porous and (**b**) non-porous.

The bacterial growth curves (Figure 8) showed that some of the tested samples were bacteriostatic, i.e., inhibited the proliferation of bacteria [54], whereas in the presence of the other ones, this process was unaffected or only slightly affected. Thus, a bacteriostatic effect towards both *S. aureus* and *E. coli* bacteria was observed for all the modified materials in which M_2_^Vi^ was used as a cross-linker (P_M/Naa, P_M/Naa/BnCl, P_M/Naa/OcBr and NP_M/Naa, NP_M/Naa/BnCl, NP_M/Naa/OcBr samples). Most modified networks containing D_4_^Vi^ moieties (P_D/Naa, P_D/Naa/BnCl, P_D/Naa/OcBr and NP_D/Naa, NP_D/Naa/BnCl samples) did not exhibit a bacteriostatic effect against either *S. aureus* or *E. coli*. NP_D/Naa/OcBr was the only exception in this group—it was bacteriostatic towards both bacteria strains studied. These results clearly demonstrated the influence of the cross-linker applied in the preparation of PHMS networks on the antibacterial properties of the modified materials.

**Figure 9:**
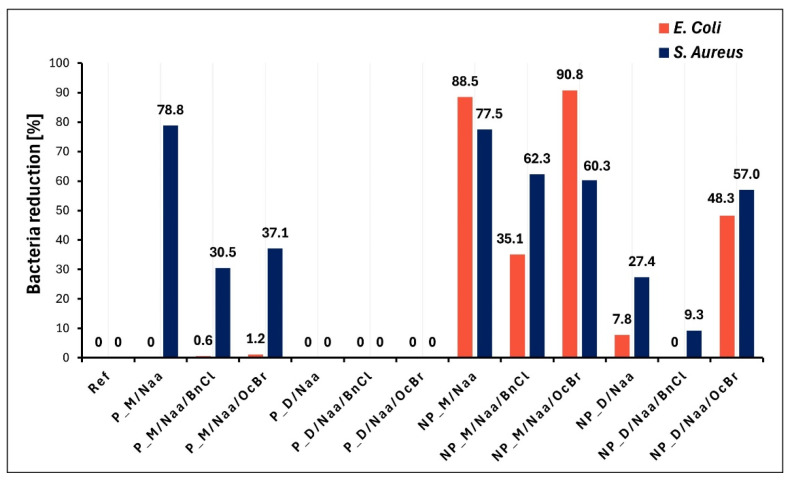
*Escherichia coli* and *Staphylococcus aureus* reduction by contact with tested materials after 24 h of incubation.

Examination of the final optical density values (OD_600_) measured in the experiments (Figure 9) revealed that the strongest bacteriostatic effect (i.e., the lowest OD_600_), again towards both *S. aureus* and *E. coli*, was demonstrated by the NP_M/Naa material (*S. aureus*: OD_600_ = 0.061, *E. coli*: OD_600_= 0.072). Treatment with BnCl or OcBr affected the antibacterial activity of the Naa-functionalized materials in different ways. The most spectacular, advantageous influence of OcBr treatment was observed for the NP_D/Naa material. It was inactive towards *S. aureus* and *E. coli*, but after reaction with OcBr, it inhibited the growth of both types of bacteria (Figure 8).

Experiments in which bacterial reduction degrees (R) were determined based on comparison with the reference sample (Section 3.7) confirmed most of the results of bacterial growth studies. Importantly, among the studied samples, the highest antibacterial effect was exhibited by the NP_M/Naa and NP_M/Naa/OcBr samples against Gram-negative *E. coli* (Figure 9). The high reduction degrees found for these materials (R = 88.5% and 90.8%, respectively, Figure 9) allowed us to consider them bactericidal towards *E. coli.*

From the conducted experiments, the antibacterial activity of the materials containing neutral Naa groups was evident even though it is generally accepted that for effective interactions with bacteria, positively charged ammonium groups in amine bactericides are required [11]. However, it was shown that uncharged, *t*-butylamine substituted siloxane copolymers were active by contact against Gram-positive and Gram-negative bacteria [31]. The operating mechanism was not fully understood; it seemed to be complex as no adsorbed bacteria on the surface of bactericidal polymers were observed by SEM [31].

Additionally, our antibacterial tests clearly showed that more investigated materials exhibited an effect on the Gram-positive bacteria *S. aureus* than on Gram-negative *E. coli* (9 vs. 5 samples, respectively, Figure 9). Higher activity against Gram-positive than Gram-negative bacteria was reported also for other amine/ammonium group-modified siloxane polymers [29,31,32,33,34]. In all cases, this difference was explained by structural differences in cell walls of these bacteria. Gram-positive bacterial cells have a loosely packed peptidoglycan layer, while cell walls of Gram-negative bacteria are composed of two membranes (outer and inner) with an additional layer of lipopolysaccharides (LPSs) [10]. Macromolecular biocides, whose bactericidal effect is due to their disruptive interaction with the bacterial cell wall and/or cytoplasmic membranes [55], can interact more effectively with Gram-positive bacterial cells. Cell walls of Gram-negative bacteria serve as barriers against external agents, making them less sensitive to environmental factors [55].

Attempting to rationalize the results of the performed antibacterial studies, the role of three key parameters in which our materials differed, namely functionalization degree by Naa, network cross-linking density and microstructure, should be considered. It should also be taken into account that the tested samples did not swell in aqueous media (verified by swelling experiments in nutrient broth performed in the conditions of antibacterial tests). Therefore, it could be supposed that in the interactions with bacteria, only their surface was involved. This precluded the contribution of free amine trapped in the bulk of the NP_D/Naa sample (Section 2.2) to the antibacterial activity of this material. Similarly, the reactions with BnCl and OcBr proceeded on the surface of Naa-functionalized materials that did not swell in BnCl and OcBr solutions in acetone applied in the conversion of amino to ammonium groups (Section 3.5). In this way, the possibility of the formation of low-molecular-weight Naa/BnCl or Naa/OcBr salts (after reaction with free Naa) that could leach to the environment was eliminated. Hence, all the observed antibacterial properties could be attributed to the studied functionalized materials and should be treated as their inherent feature.

The highest activity of the NP_M/Naa sample against *E. coli* bacteria (not significantly increased by OcBr treatment) and relatively high activity against *S. aureus* bacteria (Figure 9) could be due to its highest functionalization degree and lowest cross-linking density revealed by ^29^Si MAS-NMR spectroscopy and swelling experiments (Table 3, NMR: the lowest share of T units). Bioactivity of functionalized linear, uncross-linked polysiloxanes is believed to be enhanced by the high flexibility of their chains, which allows polymer molecules to adopt conformations that ensure efficient interactions with bacteria cell walls [30,34]. In the lightly cross-linked NP_M/Naa network, flexibility of siloxane chains could be partially preserved.

On the other hand, high activity of the P_M/Naa sample towards *S. aureus* bacteria (Figure 9) could be mainly related to the highest cross-linking degree of the P_M network found by ^29^Si MAS-NMR spectroscopy (Table 2, the highest share of T units), which meant that functionalization occurred predominantly on the surface of this material. It can be supposed that—even though the functionalization degree of the P_M/Naa material was not high (Table 3)—biocidal moieties were easily accessible for contact with bacteria, facilitated also by the porous structure of this sample.

Finally, a significant increase in the activity of the NP_D/Naa material treated with OcBr against both investigated bacteria strains (Figure 9) indicated that in this case, the presence of ammonium groups containing a long alkyl chain was necessary. Presumably, as suggested by moderate R values (Figure 9), the amount of ammonium groups in the NP_D/Naa/OcBr sample was too low to provide high bioactivity. An antibacterial effect is known to occur after a certain threshold content of ammonium groups in the polymer is attained (e.g., 20% for polyurethanes [23] and 50% for polymethacrylates [13] modified by QAS were reported).

In summary, the antibacterial properties of the studied PHMS networks modified by Naa reflected the interplay between their functionalization degrees, cross-linking levels and microstructure. The highest antibacterial activity showed the non-porous material of the highest functionalization degree and the lowest cross-linking degree. Porosity seemed to facilitate access of biocidal moieties to bacteria in the highly cross-linked material of a lower functionalization degree, which resulted in enhanced antibacterial activity.

## 3. Materials and Methods

### 3.1. Materials

Trimetylsilyl-terminated poly(hydromethylosiloxane) (PHMS, average molecular weight: 3600 g/mol), 1,1,3,3-tetramethyl-1,3-divinyldisiloxane (M_2_^Vi^), 1,3,5,7-tetramethyl-1,3,5,7-tetravinylcyclotetrasiloxane (D_4_^Vi^), Karstedt’s catalyst solution (2 wt.% of Pt in xylene) and non-ionic surfactant dimethylsiloxane-ethylene glycol copolymer 25–30% (DBE-224) were purchased from ABCR (Karlsruhe, Germany). Before application in the preparation of the non-porous materials (Section 3.3), PHMS was dried on a vacuum line at the temperature of 60 °C for 4 h. N-allylaniline (Naa) was purchased from Merck (Poznań, Poland) and purified by vacuum distillation before use. Benzyl chloride (BnCl) and 1-bromooctane (OcBr) were also bought from Merck (Poznań, Poland) and applied in the studies without any preliminary treatment. The solvents toluene, acetone and chlorobenzene were supplied by Avantor (Gliwice, Poland) and purified before use by standard procedures [56].

### 3.2. Preparation of the Porous Materials (P_M and P_D)

Porous materials (polyHIPEs) were obtained by cross-linking of PHMS with M_2_^Vi^ or D_4_^Vi^ in water-in-oil (w/o) HIPE conditions according to the method reported in Refs. [38,57]. The general procedure involved the formation of the continuous oil phase of the emulsion in the first stage. PHMS, M_2_^Vi^ or D_4_^Vi^, DBE-224 and chlorobenzene (porogen) were thoroughly mixed in a glass vial. To the obtained mixture, under constant stirring, Karstedt’s catalyst solution was introduced. Then, the oil phase was placed in an agate mortar and the internal aqueous phase (0.02 M NaCl solution in water) was added dropwise with constant stirring. The resulting homogeneous emulsion was transferred into a Teflon crucible, placed in an oven and heated at 75 °C for 24 h. Monolithic material obtained after this time was cut into small (few millimeters in each size) pieces and extracted with acetone in a Soxhlet apparatus for 12 h. Finally, the obtained foam was dried in air at room temperature.

In a typical polyHIPE preparation process, 1.0 g of PHMS (0.263 mmol; 0.016 mol of Si–H groups) was used, while the amount of M_2_^Vi^ (0.249 g; 1.333 mmol) and D_4_^Vi^ (0.230 g; 0.668 mmol) was calculated to obtain a molar ratio of Si-H groups from the polymer to Si-Vi groups from a crosslinking agent equal to 1:0.17. The amounts of other HIPE components were as follows: DBE-224 constituted 20% of the sum of PHMS and M_2_^Vi^ weights (0.315 g) or PHMS and D_4_^Vi^ weights (0.308 g) and chlorobenzene constituted 35% of the sum of the PHMS, M_2_^Vi^ and DBE-224 weights (0.842 g) or 30% of the sum of the PHMS, D_4_^Vi^ and DBE-224 weights (0.659 g), whereas NaCl solution constituted 74 wt% of the emulsion (6.9 mL and 6.2 mL in the reactions with M_2_^Vi^ and D_4_^Vi^, respectively). In the reactions, 7 μL (6.39∙10^−7^ mol of Pt) of Karstedt’s catalyst solution was used.

### 3.3. Preparation of the Non-Porous Materials (NP_M and NP_D)

To prepare the non-porous materials, cross-linking of PHMS by hydrosilylation of M_2_^Vi^ or D_4_^Vi^ was performed in the bulk. The molar ratio of Si–H groups to Si–Vi groups in the reaction was equal to 1:0.17. In a typical cross-linking experiment, the mixture of 1.0 g (0.263 mmol; 0.016 mol of Si–H groups) of PHMS (previously dried on a vacuum line, Section 3.1), the appropriate amount of M_2_^Vi^ (0.249 g; 1.333 mmol) or D_4_^Vi^ (0.230 g; 0.668 mmol) and Karstedt’s catalyst solution (5 µL; 4.53∙10^–7^ mol of Pt) were successively placed in a Schlenk flask and then heated at 60 °C for 24 h in Ar atmosphere. After the reaction, the obtained non-porous material was dried on a vacuum line at 60 °C.

### 3.4. Functionalization of Porous and Non-Porous Materials by Naa

Porous and non-porous materials were functionalized with Naa by hydrosilylation performed in the presence of Karstedt’s catalyst. The molar ratio of Si–H groups theoretically remaining in the material after the cross-linking process to CH_2_=CH-CH_2_- groups from Naa equal to 1:1 was applied for all porous and non-porous materials.

In a typical run, 1.0 g (0.013 mol of Si-H groups susceptible to functionalization) of the solid polymer material (P_M, P_D, NP_M or NP_D), the measured amounts of Naa (1.763 mL, 0.013 mol), toluene (14 mL and 10 mL in porous and non-porous sample modification, respectively) and Karstedt’s catalyst solution (7 µL and 5 µL in porous and non-porous sample modification, respectively) were successively placed in the flowing Ar atmosphere in a Schlenk flask. Then, the flask was closed, placed in an oil bath, and its content, under magnetic stirring, was heated to 60 °C. The reaction was carried out at this temperature for 72 h. After this time, materials were washed a few times with anhydrous toluene on a Büchner funnel in order to remove all unreacted amine from the sample. Then, the obtained products (P_M/Naa, P_D/Naa and NP_M/Naa, NP_D/Naa) were dried on a vacuum line.

### 3.5. Reaction of Functionalized Materials with BnCl and OcBr

Functionalized materials were reacted with BnCl and OcBr in order to transform the amine groups present in the materials into ammonium groups.

As in a typical procedure, 1.0 g of the functionalized material, 10 mL of acetone and 4.603 mL of BnCl (0.040 mol; a threefold molar excess to the theoretical amount of amino groups introduced into the polymer) were placed in a round-bottom flask and refluxed for 72 h with constant stirring. Then, the sample was washed a few times with acetone on a Büchner funnel in order to remove all unreacted BnCl. The obtained product (P_M/Naa/BnCl, P_D/Naa/BnCl, NP_M/Naa/BnCl or NP_D/Naa/BnCl) was dried on a vacuum line.

The reactions with OcBr (resulting in P_M/Naa/OcBr, P_D/Naa/OcBr, NP_M/Naa/OcBr or NP_D/Naa/OcBr) were conducted analogously using 6.909 mL of OcBr (0.040 mol).

### 3.6. Characterization Methods

Equilibrium swelling of the prepared polyHIPEs and non-porous materials was determined in toluene. To the weighed amount of the studied sample, an excess of the solvent was added. After 36 h, the excess solvent was separated and the swollen sample was weighed. Swelling degrees reported in this work were calculated as follows: (m_s_ − m_0_)/m_0_ ratios, where m_s_ is the weight of the swollen sample and m_0_—is the weight of the sample subjected to swelling.

Skeletal density (d_sk_) of all the samples was measured using a Micromeritic AccuPyc II 1340 helium pycnometer. The apparent (bulk) density (d_app_) of porous samples was determined by weighing cuboids 5 × 5 × 4 mm (length × breadth × height) in size cut from the samples. Total porosity of the prepared materials given in the work was calculated by the following equation: (d_sk_ − d_app_/d_sk_) × 100%.

Scanning electron microscopy (SEM) studies were performed using a Phenom XL (Thermo Fisher Scientific, Waltham, MA, USA) microscope. SEM micrographs were also applied to evaluate the diameters of pores present in the P_M and P_D materials. They were determined using the ImageJ 1.53k software by the procedure presented in Ref. [28]. In the analyses, 100 voids and 200–300 windows were taken into account. Based on the results, mean diameters with standard deviations and median diameters with interquartile ranges were calculated. To verify the statistical significance of differences in the observed pore diameters, a nonparametric Mann–Whitney U-test [58] was used.

FTIR spectra were recorded on a Vertex 70v spectrometer. Spectra were measured in the range of 4000 to 400 cm^−1^, in the transmission mode using a standard KBr pellet technique. The resolution of the measurements was equal to 4 cm^−1^. Spectra analyses were conducted in the Opus 7.2. program after correcting their baselines. Quantitative analysis involved calculating the ratios of integral intensities of the band due to Si-H bond stretching vibrations at ~2162 cm^−1^ and symmetric bending vibrations of the Si-CH_3_ group at ~ 1263 cm^−1^ in the FTIR spectra of the obtained materials. Since Si-CH_3_ groups do not participate in the hydrosilylation reaction, the intensity of this FTIR band remained unchanged in the spectra. The Si-H/Si-CH_3_ band area ratios found for the starting materials (PHMS in the case of cross-linking reactions or cross-linked products in the case of functionalization) were assumed to represent 100% and the corresponding ratios for the final reaction products were expressed as a percentage of this initial value given in the work as conversion degrees of Si-H groups.

Elemental analyses were taken on a Vario El III analyzer (Elementar Analysensysteme GmbH, Langenselbol, Germany) after combustion of the analyzed sample in oxygen at 1150 °C. Contents of C, H and N in the samples reported in the work are the average values of two analyses. The contents of Si and O in the samples were calculated as the difference 100%–ΣC,H,N.

^29^Si MAS-NMR spectra of the samples were measured in a single pulse excitation (SPE) mode on a Bruker (Billerica, MA, USA) Avance III 500 MHz WB (11.7 T) spectrometer operating at a frequency of 99.4 MHz. The samples were packed into 4.0 mm rotors and a Bruker HP-WB highspeed MAS probe was used with a spinning frequency of 8 kHz. A π/3 pulse (6.0 µs) and the repetition time of 31 s were used. Typically, 4200 scans were acquired. Deconvolutions of all NMR spectra were performed using the Origin 2023 program (OriginLab Corporation, MA, USA).

X-ray photoelectron spectroscopy (XPS) measurements were carried out with a hemispherical analyzer (SES R4000, Gammadata Scienta, Uppsala, Sweden), The unmonochromatized MgKa X-ray source with the anode operating at 12 kV and 20 mA current emissions was applied to generate core excitation. All binding energy values were corrected to the carbon C1s excitation at 285.0 eV. The samples were pressed into indium foil and mounted on a holder. All spectra were collected at a pass energy of 100 eV, except survey scans which were collected at a pass energy of 200 eV. Intensities were estimated by calculating the 12467 integral of each peak, after subtraction of the Shirley-type background, and fitting the experimental curve with a combination of Gaussian and Lorentzian lines of fixed proportions (70:30).

### 3.7. Antibacterial Tests

Two commercial bacterial strains, Gram-negative (*Escherichia coli* ATCC 8739) and Gram-positive (*Staphylococcus aureus* ATCC 6538P), were used to evaluate the antimicrobial activity of the modified polysiloxane materials. The effect of the powdered materials on the bacterial growth kinetics in liquid media was quantitatively studied. The study was conducted according to the procedure described in Refs. [59,60]; 1 mg of each sample was placed in the wells of a culture plate in nutrient broth and phosphate buffer followed by the addition of bacteria at a concentration of ~10^5^ CFU/mL. Bacterial growth rates were determined by measuring the optical density at 600 nm (OD_600_) using a UV–Vis spectrometer (Shimadzu 2600i). The measurements were carried out at specific time intervals for 24 h. Bacterial growth curves were obtained by plotting the optical density as a function of incubation time.

In the second step of the experiment, the degree of reduction in bacteria (*E. coli* and *S. aureus*) was determined. As a method, the protocol of Ref. [61] was used. Bacterial cultures were refreshed twice before analysis by transferring onto nutrient agar slants and incubated for 24 h at 35 °C. Microbial cells were resuspended in sterile nutrient broth approximately 10^5^ CFU/mL. The prepared test powders with the same weight (15 mg) were placed in sterile dishes. Next, 1.5 mL of the inoculum was applied to each sample and placed in a culture well. The test and control materials were incubated for 24 h at 35 °C. From each SCDLP solution (for test and control), 0.5 mL was taken and suspended in 4.5 mL of buffered saline solution. After thorough homogenization, further decimal dilutions of the inoculum were prepared. From each sample of the wash solution and serial dilution, 0.1 mL of the suspension was applied to the surfaces of sterile Plate Count Agar (PCA). All plates with dilutions spread on the agar were placed in an incubator and incubated (24 h, 35 °C temperature). At the end of the incubation, the cells grown on the plates were counted. The bacterial reduction (*R*) was calculated according to the following Equation (1):

(1) $$R\%=\left(\frac{\mathrm{concentration}\;A-\mathrm{concentration}\;B}{\mathrm{concentration}\;A}\right)\cdot 100\%$$

where concentration *A*—number of bacteria CFU/mL for the tested sample (with the material sample) after 24 h incubation; concentration *B*—number of bacteria CFU/mL for the reference (without the material sample) after 24 h incubation.

## 4. Conclusions

The studies presented in the work show that it is possible to prepare Naa-functionalized PHMS networks by a simple hydrosilylation reaction between Si-H groups preserved in the relevant systems and Naa. The Naa-modified materials thus obtained exhibit attractive antibacterial properties, which can be further modulated by treatment with BnCl or OcBr. It should be pointed out that Naa-functionalized PHMS networks are new materials that have never been described in the literature before. In our opinion, their investigations in the present work will be helpful for future developments in the field of antibacterial polysiloxanes.

## Figures and Tables

**Table 1:** Characterization of the prepared polyHIPEs based on SEM image analysis.

Sample	Diameter Range [μm]		Main Fraction: Diameter Range [μm] (Share [%])		Mean (Median) Diameter [μm]	
	Voids	Windows	Voids	Windows	Voids	Windows
P_M	1.4–6.1	0.6–1.6	1.4–4.8 (97)	0.6–1.4 (97)	3.0 ± 0.89 (2.7 ± 1.06)	0.8 ± 0.24 (0.7 ± 0.35)
P_D	1.1–5.6	0.4–1.3	1.2–3.2 (95)	0.4–1.2 (98)	2.2 ± 0.7 (1.9 ± 0.64)	0.8 ± 0.18 (0.7 ± 0.21)

**Table 2:** Characterization of the starting materials.

Sample	Conversion Degree of Si-H Groups *^a^* [%]	^29^Si MAS-NMR: Chemical Shift of the Signal *^b^* [ppm] (Share [%])					Swelling Degree [g/g]	Density [g/cm^3^]		Total Porosity [%]
		M Units	D Units	D^H^ Units	D^OH^ Units	T Units		Skeletal	Apparent	
P_M	94.8	8.1 (15)	−20.0; −22.2 (5; 10)	−35.2; −36.4; −37.6 (5)	−56.4 (22)	−65.8 (43)	2.7	1.2882 ± 0.0022	0.2458	80.9
P_D	88.5	-	−19.1; −22.2 (28; 8)	−34.0; −35.2; −37.1 (6)	−56.0 (25)	−65.4 (33)	5.1	1.3538 ± 0.0104	0.1442	89.4
NP_M	62.0	8.0 (16)	−19.0; −20.5 (6; 10)	−34.0; −34.3; −34.6 (63)	−56.1 (1)	−65.8 (4)	1.2	1.0998 ± 0.0037	-	-
NP_D	58.3	9.8 (1)	−19.7 (29)	−34.6; −36.3 (50)	−56.3 (4)	−65.9 (16)	0.6	1.1494 ± 0.0037	-	-

*^a^* Calculated by dividing the ratios of integral intensities of Si–H (at 2169 cm^−1^) and Si-CH_3_ (at 1261 cm^−1^) bands in the FTIR spectra of PHMS and cross-linked products. *^b^* Symbols denote the following: M—[SiO(CH_3_)_2_(CH_2_CH_2_)] units, D—[SiO_2_(CH_3_)(CH_2_CH_2_)], [SiO_2_(CH_3_)(CH(CH_3_))] units, D^H^—[SiO_2_(CH_3_)H] units, D^OH^—[SiO_2_(CH_3_)OH] units; T—[SiO_3_(CH_3_)] units.

**Table 3:** Characterization of porous and non-porous materials modified by Naa.

Sample	Contents of Elements [wt.%]					Conversion Degree of Si-H Groups *^d^* [%]	^29^Si MAS-NMR: Chemical Shift of the Signal *^e^* [ppm] (Share [%], Δ-Share Difference with Respect to the Starting Spectrum)					Swelling Degree [g/g]	Density [g/cm^3^]		Total Porosity [%]
	N		C	H	SiO *^c^*										
	Calc. *^a^*	Found *^b^*					M Units	D Units	D^H^ Units	D^OH^ Units	T Units		Skeletal	Apparent	
P_M/Naa	5.72	0.96 (16.70)	29.54	6.29	63.22	4.3	8.3 (15, Δ = 0)	−20.3; −22.2 (19, Δ = +4)	−35.9 (2, Δ = −3)	−56.5 (21, Δ = −1)	−65.6 (43, Δ = 0)	3.6	2.0014 ±0.2020	0.1502	92.5
P_D/Naa	5.07	0.31 (6.11)	25.21	6.49	67.89	3.5	-	−19.8; −22.4 (37, Δ = +1)	−37.2 (8, Δ = +2)	−56.8 (17, Δ = −8)	−65.5 (39, Δ = +6)	5.3	1.3220 ±0.0087	0.1924	85.4
NP_M/Naa	5.72	1.58 (27.69)	36.84	7.60	53.98	8.0	8.0 (16, Δ = 0)	−19.0; −20.8 (27, Δ = +11)	−34.6; −36.8 (47, Δ = −16)	- (Δ = −1)	−65.6 (10, Δ = +6)	1.4	1.2473 ±0.0265	-	-
NP_D/Naa	5.07	3.14 (61.93)	38.01	6.77	52.08	7.2	9.4 (2, Δ = 0)	−20.2 (34, Δ = +5)	−34.9; −36.4 (49, Δ = −1)	−56.2 (2, Δ = −2)	−65.8 (13, Δ =−3)	0.6	1.1701 ±0.0031	-	-

*^a^* The maximum possible nitrogen content [%] resulting from the stoichiometry of the reaction. *^b^* In brackets: fractions of N incorporated into the polymer, calculated as (N_found_/N_calc_.)·100%. *^c^* %SiO = 100–Σ %C, % H, %N. *^d^* Calculated by dividing the ratios of integral intensities of Si–H (at ~2162 cm^−1^) and Si-CH_3_ (at ~1260 cm^−1^) bands in the FTIR spectra of starting materials and corresponding functionalized products. *^e^* Symbols denote the following: M—[SiO(CH_3_)_2_(CH_2_CH_2_)] units, D—[SiO_2_(CH_3_)(CH_2_CH_2_)], [SiO_2_(CH_3_)(CH(CH_3_))] units, D^H^—[SiO_2_(CH_3_)H] units, D^OH^—[SiO_2_(CH_3_)OH] units; T—[SiO_3_(CH_3_)] units.

## Data Availability

The original contributions presented in this study are included in the article/Supplementary Material. Further inquiries can be directed to the corresponding author.

## Supplementary Materials

The following supporting information can be downloaded at https://www.mdpi.com/article/10.3390/ijms26146700/s1.

## References

[1] Lawrence S.A. (2004). Amines: Synthesis, Properties and Applications.

[2] Barraza R., Sertage A.G., Kajjam A.B., Ward C.L., Lutter J.C., Schlegel H.B., Allen M.J. (2022). Properties of Amine-Containing Ligands That Are Necessary for Visible-Light-Promoted Catalysis with Divalent Europium. Inorg. Chem..

[3] Narzary B.B., Baker B.C., Yadav N., D’Elia V., Faul C.F.J. (2021). Crosslinked porous polyimides: Structure, properties and applications. Polym. Chem..

[4] Grzęda D., Węgrzyk G., Nowak A., Komorowska G., Szczepkowski L., Ryszkowska J. (2023). Effect of Different Amine Catalysts on the Thermomechanical and Cytotoxic Properties of ‘Visco’-Type Polyurethane Foam for Biomedical Applications. Materials.

[5] Tariq Aziz T., Haq F., Farid A., Cheng L., Chuah L.F., Bokhar A., Mubashir M., Tang D.Y.Y., Show P.L. (2024). The epoxy resin system: Function and role of curing agents. Carbon Lett..

[6] Oldenhove de Guertechin L., Barel A.O., Paye M., Maibach H.I. (2009). Surfactants: Classification. Handbook of Cosmetic Science and Technology.

[7] Florek J., Guillet-Nicolas R., Kleitz F., Leclerc M., Gauvin R. (2014). Ordered mesoporous silica: Synthesis and applications. Functional Materials: For Energy, Sustainable Development and Biomedical Sciences.

[8] Makosza M. (2000). Phase-transfer catalysis. A general green methodology in organic synthesis. Pure Appl. Chem..

[9] Walker E.B., Paulson D.S. (2002). Quaternary Ammonium Compounds. Handbook of Topical Antimicrobials. Industrial Applications in Consumer Products and Pharmaceuticals.

[10] Kwaśniewska D., Chen Y.-L., Wieczorek D. (2020). Biological Activity of Quaternary Ammonium Salts and Their Derivatives. Pathogens.

[11] Grigoras A.G. (2021). Natural and synthetic polymeric antimicrobials with quaternary ammonium moieties: A review. Environ. Chem. Lett..

[12] Guo J., Qin J., Ren Y., Wang B., Cui H., Ding Y., Mao H., Yan F. (2018). Antibacterial activity of cationic polymers: Side-chain or main-chain type?. Polym. Chem..

[13] Palermo E.F., Kuroda K. (2009). Chemical Structure of Cationic Groups in Amphiphilic Polymethacrylates Modulates the Antimicrobial and Hemolytic Activities. Biomacromolecules.

[14] Kuroda K., Caputo G.A., DeGrado W.F. (2009). The Role of Hydrophobicity in the Antimicrobial and Hemolytic Activities of Polymethacrylate Derivatives. Chem. Eur. J..

[15] Vigliotta G., Mella M., Rega D., Izzo L. (2012). Modulating Antimicrobial Activity by Synthesis: Dendritic Copolymers Based on Nonquaternized 2-(Dimethylamino)ethyl Methacrylate by Cu-Mediated ATRP. Biomacromolecules.

[16] Thoma L.M., Boles B.R., Kuroda K. (2014). Cationic Methacrylate Polymers as Topical Antimicrobial Agents against *Staphylococcus aureus* Nasal Colonization. Biomacromolecules.

[17] Chin W., Yang C., Ng V.W.L., Huang Y., Cheng J., Tong Y.W., Coady D.J., Fan W., Hedrick J.L., Yang Y.Y. (2013). Biodegradable Broad-Spectrum Antimicrobial Polycarbonates: Investigating the Role of Chemical Structure on Activity and Selectivity. Macromolecules.

[18] Fukushima K., Kishi K., Saito K., Takakuwa K., Hakozaki S., Yano S. (2019). Modulating bioactivities of primary ammonium tagged antimicrobial aliphatic polycarbonates by varying length, sequence and hydrophobic side chain structure. Biomater. Sci..

[19] Nimmagadda A., Liu X., Teng P., Su M., Li Y., Qiao Q., Khadka N.K., Sun X., Pan J., Xu H. (2017). Polycarbonates with Potent and Selective Antimicrobial Activity toward Gram-Positive Bacteria. Biomacromolecules.

[20] Biying A.O., Ong M.J.H., Zhao W., Li N., Luo H.-K., Chan J.M.W. (2024). Broad-Spectrum Antimicrobial Polymer-Coated Fabrics via Commodity Polystyrene Upcycling. ACS Appl. Polym. Mater..

[21] Chang C.-H., Chang C.-H., Yang Y.-W., Chen H.-Y., Yang S.-J., Yao W.-C., Chao C. (2022). -Y. Quaternized Amphiphilic Block Copolymers as Antimicrobial Agents. Polymers.

[22] Udabe E., Isik M., Sardon H., Irusta L., Salsamendi M., Sun Z., Zheng Z., Yan F., Mecerreyes D. (2017). Antimicrobial polyurethane foams having cationic ammonium groups. J. Appl. Polym. Sci..

[23] Hu P., Greiner A., Agarwal S. (2019). Synthesis and Properties Evaluation of Quaternized Polyurethanes as Antibacterial Adhesives. J. Polym. Sci. Part A Polym. Chem..

[24] Wang Y., Chen R., Li T., Ma P., Zhang H., Du M., Chen M., Dong W. (2020). Antimicrobial Waterborne Polyurethanes Based on Quaternary Ammonium Compounds. Ind. Eng. Chem. Res..

[25] Owińska M., Chechelska-Noworyta A., Olejniczak Z., Hasik M. (2021). Functionalization of polyhydromethylsiloxane with nitrogen-containing organic compounds. J. Polym. Res..

[26] Marciniec B., Guliński J., Urbaniak W., Kornetka Z.W. (1992). Comprehensive Handbook on Hydrosilylation.

[27] Wójcik-Bania M., Łącz A., Nyczyk-Malinowska A., Hasik M. (2017). Poly(methylhydrosiloxane) networks of different structure and content of Si-H groups: Physicochemical properties and transformation into silicon oxycarbide ceramics. Polymer.

[28] Mrówka J., Kosydar R., Gackowski M., Gurgul J., Lityńska-Dobrzyńska L., Handke B., Drelinkiewicz A., Hasik M. (2022). Poly(hydromethylsiloxane)-derived high internal phase emulsion-templated materials (polyHIPEs) containing palladium for catalytic applications. J. Mater. Sci..

[29] Mizerska U., Fortuniak W., Chojnowski J., Hałasa R., Konopacka A., Werel W. (2009). Polysiloxane cationic biocides with imidazolium salt (ImS) groups, synthesis and antibacterial properties. Eur. Polym. J..

[30] Zhang Q., Liu H., Chen X., Zhan X., Chen F. (2015). Preparation, surface properties, and antibacterial activity of a poly(dimethyl siloxane) network containing a quaternary ammonium salt side chain. J. Appl. Polym. Sci..

[31] Mizerska U., Fortuniak W., Chojnowski J., Turecka K., Konopacka A., Werel W. (2010). Antimicrobial Siloxane Statistical and Graft Copolymers Substituted with t-Butylamine and t-Butylammonium Biocidal Functions. J. Inorg. Organomet. Polym..

[32] Lin Y., Liu Q., Cheng L., Lei Y., Zhang A. (2014). Synthesis and antimicrobial activities of polysiloxane-containing quaternary ammonium salts on bacteria and phytopathogenic fungi. React. Funct. Polym..

[33] He S., Hou M., Shan S., Li R., Yu N., Lin Y., Zhang A. (2023). Synthesis and anti-bacterial/fungal activities of amphiphilic polysiloxanes primary ammonium salts. React. Funct. Polym..

[34] Fortuniak W., Mizerska U., Chojnowski J., Basinska T., Słomkowski S., Chehimi M.M., Konopacka A., Turecka K., Werel W. (2011). Polysiloxanes with Quaternary Ammonium Salt Biocidal Functions and Their Behavior When Incorporated into a Silicone Elastomer Network. J. Inorg. Organomet. Polym..

[35] Zare M., Ghomi E.R., Venkatraman P.D., Ramakrishna S. (2021). Silicone-based biomaterials for biomedical applications: Antimicrobial strategies and 3D printing technologies. J. Appl. Polym. Sci..

[36] Krajnc P. (2002). The influence of some polymerization conditions on the morphology of poly(styrene-co-divinylbenzene) monoliths. Polimery.

[37] Krajnc P., Leber N., Štefanec D., Kontrec S., Podgornik A. (2005). Preparation and characterisation of poly(high internal phase emulsion) methacrylate monoliths and their application as separation media. J. Chromatogr. A.

[38] Mrówka J., Gackowski M., Lityńska-Dobrzyńska L., Bernasik A., Kosydar R., Drelinkiewicz A., Hasik M. (2020). Poly(methylvinylsiloxane)-based high internal phase emulsion-templated materials (polyHIPEs)-preparation, incorporation of palladium, and catalytic properties. Ind. Eng. Chem. Res..

[39] Mrówka J., Kosydar R., Kornaus K., Partyka J., Hasik M. (2024). Macroporous Poly(hydromethylsiloxane) Networks as Precursors to Hybrid Ceramics (Ceramers) for Deposition of Palladium Catalysts. Molecules.

[40] Kimmins S.D., Cameron N.R. (2011). Functional Porous Polymers by Emulsion Templating: Recent Advances. Adv. Funct. Mater..

[41] Silverstein M. (2014). Emulsion-templated porous polymers: A retrospective perspective. Polymer.

[42] Choudhury S., Fitzhenry L., White B., Connolly D. (2016). Polystyrene-co-Divinylbenzene PolyHIPE Monoliths in 1.0 mm Column Formats for Liquid Chromatography. Materials.

[43] Chauhan B.P., Sarkar A., Chahan M., Roka A. (2009). Water as green oxidant: A highly selective conversion of organosilanes to silanols with water. Appl. Organometal. Chem..

[44] Jeon M., Han J., Park J. (2012). Catalytic Synthesis of Silanols from Hydrosilanes and Applications. ACS Catal..

[45] Brook M.A. (2000). Silicon in Organic, Organometallic and Polymer Chemistry.

[46] Socrates G. (2004). Infrared and Raman Characteristic Group Frequencies.

[47] Marsmann H.C. (2011). Silicon-29 NMR. eMagRes.

[48] Januszewski R., Kownacki I., Maciejewski H., Marciniec B. (2017). An efficient catalytic and solvent-free method for the synthesis of mono-organofunctionalized 1,1,3,3-tetramethyldisiloxane derivatives. J. Organomet. Chem..

[49] Chechelska-Noworyta A., Owińska M., Hasik M. (2019). Hydrosilylation of nitrogen-containing organic compounds: Model studies. J. Organomet. Chem..

[50] Rohm K., Manas-Zloczower I., Feke D. (2019). Poly(HIPE) morphology, crosslink density, and mechanical properties influenced by surfactant concentration and composition. Colloids Surf. A.

[51] Kovacic S., Silverstein M.S. (2016). Superabsorbent, High Porosity, PAMPS-Based Hydrogels through Emulsion Templating. Macromol. Rapid Commun..

[52] Chojnowski J., Cypryk M., Jones R.G., Ando W., Chojnowski J. (2000). Synthesis of Linear Polysiloxanes. Silicon-Containing Polymers.

[53] Rouxhet P.G., Genet M.J. (2011). XPS analysis of bio-organic systems. Surf. Interface Anal..

[54] Campoccia D., Montanaro L., Arciola C.R. (2013). A review of the clinical implications of anti-infective biomaterials and infection-resistant surfaces. Biomaterials.

[55] Bragg R., Jansen A., Coetzee M., van der Westhuizen W., Boucher C., Adhikari R., Thapa S. (2014). Bacterial Resistance to Quaternary Ammonium Compounds (QAC) Disinfectants. Infectious Diseases and Nanomedicine II.

[56] Armarengo W.L.F., Chai C.L.L. (2003). Purification of Laboratory Chemicals.

[57] Grosse M.-T., Lamotte M., Birot M., Deleuze H. (2008). Preparation of microcellular polysiloxane monoliths. J. Pol. Sci. Part A Polym. Chem..

[58] Corder G.W., Foreman D.I. (2009). Nonparametric Statistics for Non-Statisticians: A Step-by-Step Approach.

[59] Chen S., Guo Y., Chen S., Yu H., Ge Z., Zhang X., Zhang P., Tang J. (2012). Facile preparation and synergistic antibacterial effect of three-component Cu/TiO2/CS nanoparticles. J. Mater. Chem..

[60] Wang J., Gong X., Hai J., Li T. (2018). Synthesis of silver-hydroxyapatite composite with improved antibacterial properties. Vacuum.

[61] (2007). Plastics—Measurement of Antibacterial Activity on Plastics Surfaces.

