# Supplementary material for: Poly(hydromethylsiloxane) Networks Functionalized by N-allylaniline
Source: Int J Mol Sci. 2025 Jul 12;26(14):6700. doi: 10.3390/ijms26146700 (PMC12296161; doi:10.3390/ijms26146700)
Supplement: Supplementary file 1 [file ijms-26-06700-s001.zip › ijms-3634947-supplementary.pdf]

Poly(hydromethylsiloxane) networks functionalized by N-allylaniline

Anita Wysopal[1], Maria Owińska[1], Ewa Stodolak-Zych[1], Mariusz Gackowski[2], Magdalena Hasik[1*]

[1] Faculty of Materials Science and Ceramics, AGH University of Krakow, Kraków, Poland
[2] Jerzy Haber Institute of Catalysis and Surface Chemistry Polish Academy of Sciences, Kraków, Poland
* Correspondence: mhasik@agh.edu.pl

SUPPORTING INFORMATION

Contents:

Figure S1. Distribution plots of void and window diameters in the P_D and P_M polyHIPEs determined by the analysis of SEM images.

Figure S2. SEM images of the functionalized materials.

Figure S3. FTIR spectra of the final, porous and non-porous Naa functionalized materials after modification by BnCl or OcBr.

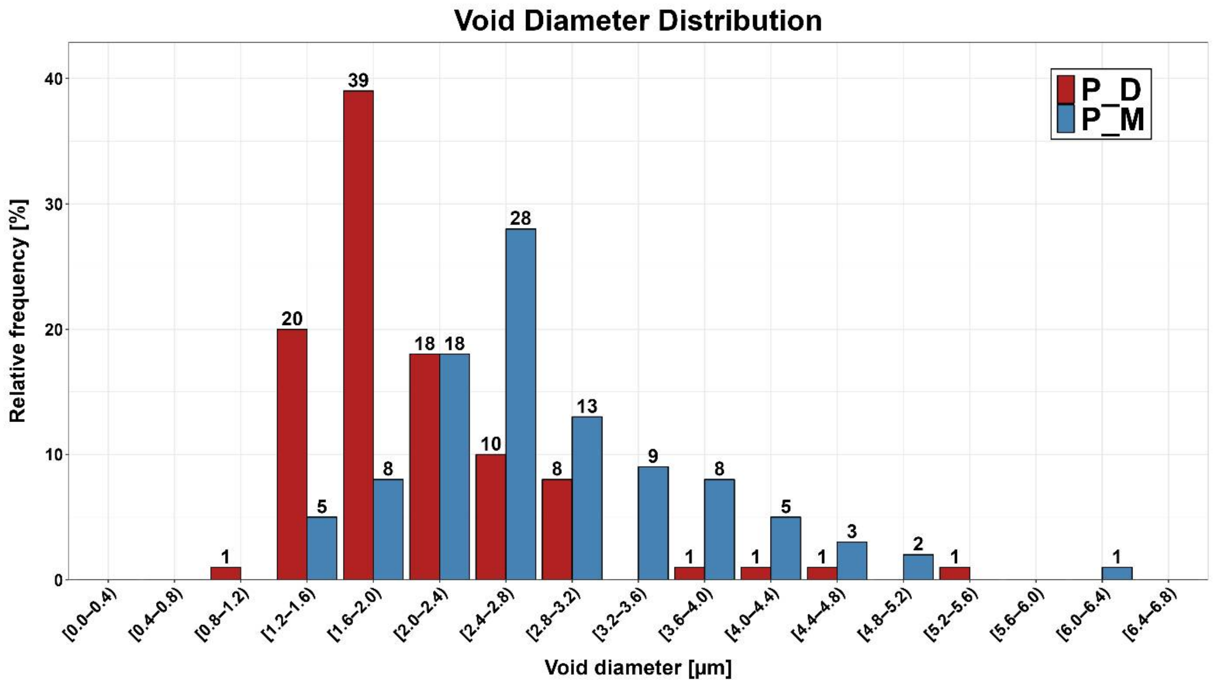

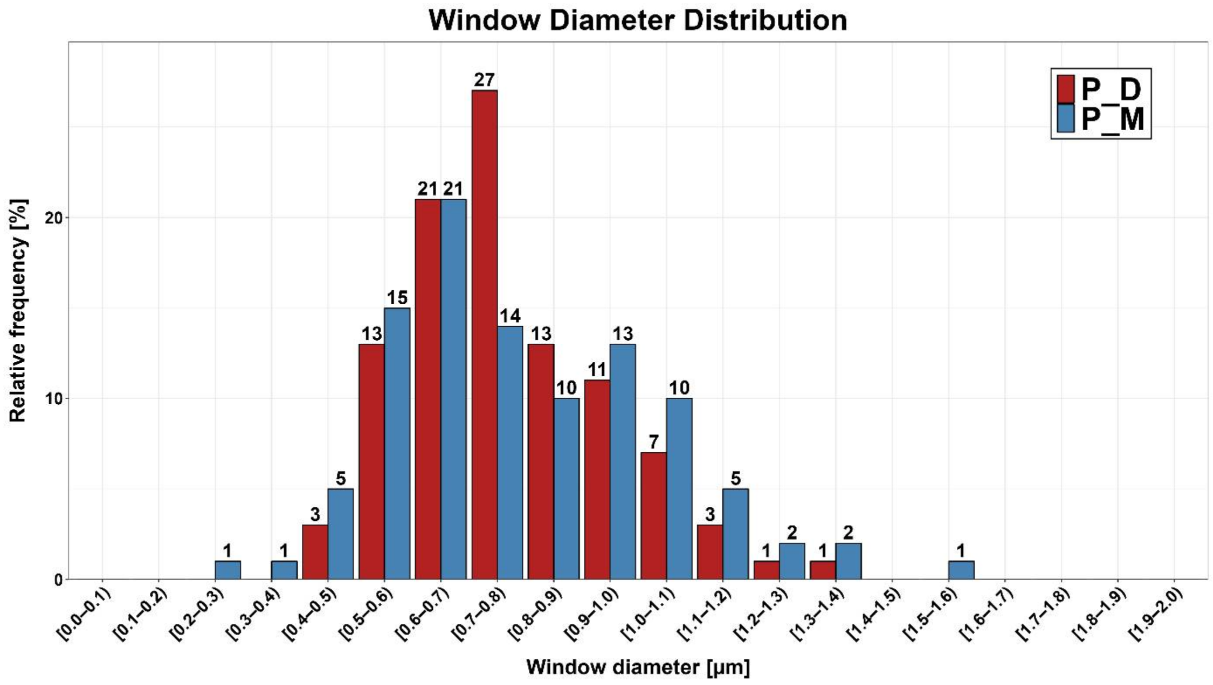

Figure S1. Distribution plots of void and window diameters in the P_D and P_M polyHIPEs determined by the analysis of SEM images.

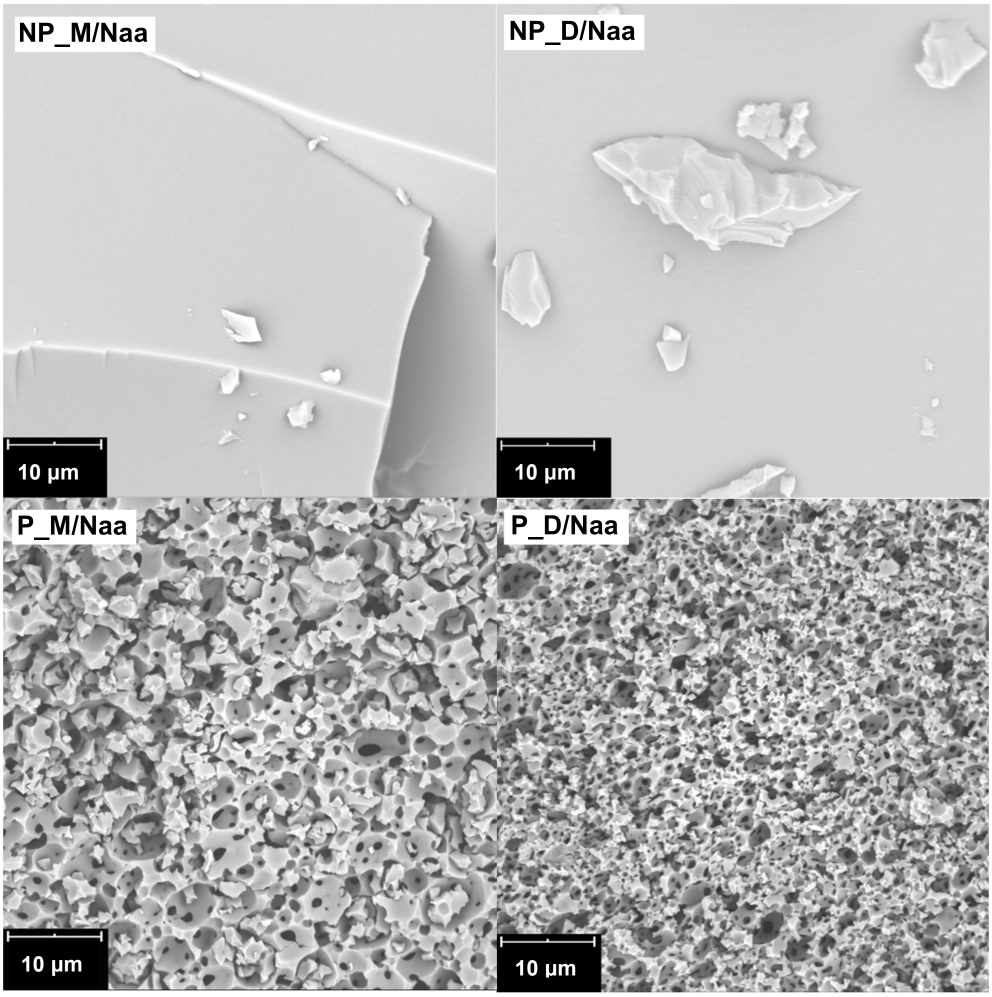

Figure S2. SEM images of the functionalized materials.

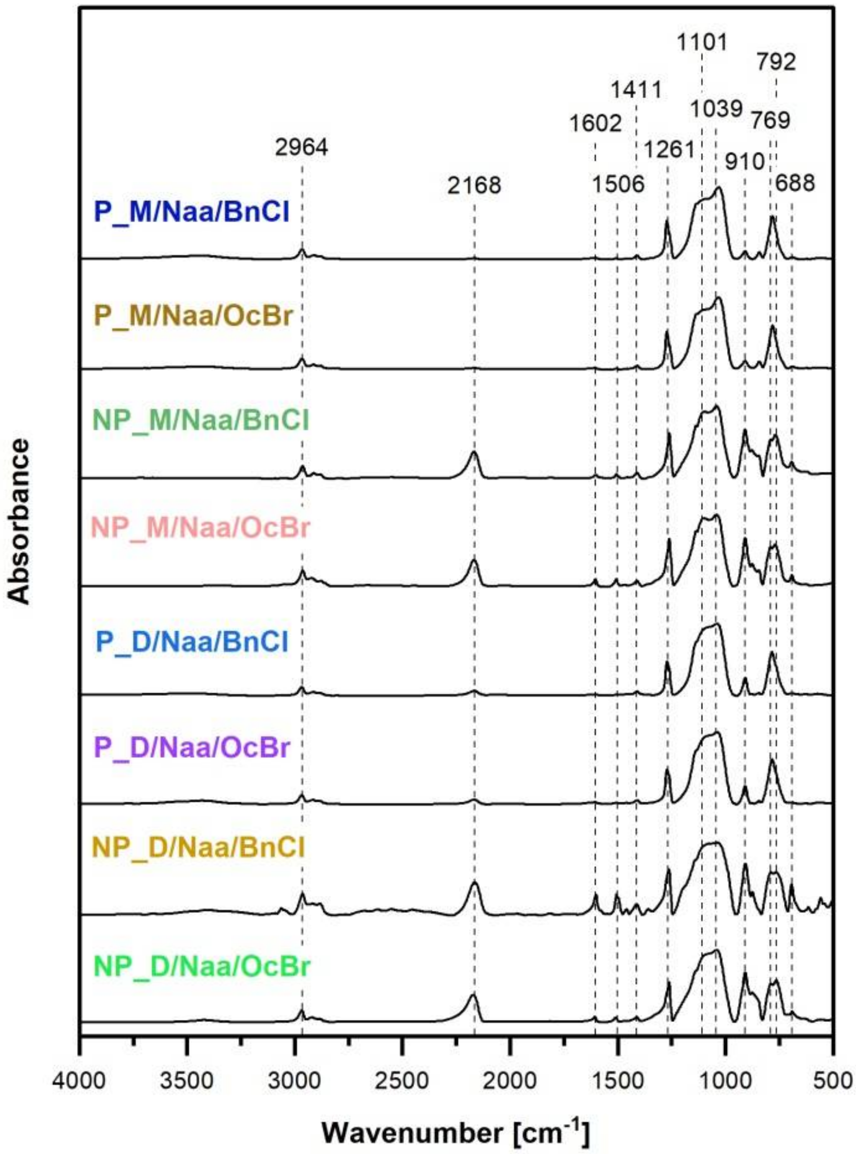

Figure S3. FTIR spectra of the final, porous and non-porous Naa functionalized materials after modification by BnCl or OcBr.
